# A multiplex interactome of Ebola virus proteins reveals TM9SF2 as a cell‐surface attachment factor that promotes viral entry

**DOI:** 10.1002/imo2.70116

**Published:** 2026-07-02

**Authors:** Limin Shang, Shouwen Du, Yuchen Liu, Letian Li, Xi Wu, Yanzhi Yuan, Chaozhi Jin, Qingkun Mi, Yuhang Wang, Xiaoshuang Shi, Jiamin Wang, Guoqing Zhang, Yidan Ma, Weijin Huang, Jigang Wang, Jian Wang, Chang Li

**Affiliations:** ^1^ State Key Laboratory of Medical Proteomics National Center for Protein Sciences (Beijing) Beijing China; ^2^ Key Laboratory of Livestock Disease Prevention of Guangdong Province, Key Laboratory for prevention and control of Avian Influenza and Other Major Poultry Diseases, Ministry of Agriculture and Rural Affairs, Institute of Animal Health Guangdong Academy of Agricultural Sciences Guangzhou China; ^3^ Department of Infectious Diseases, Shenzhen People's Hospital, The First Affiliated Hospital Southern University of Science and Technology Shenzhen China; ^4^ State Key Laboratory of Pathogen and Biosecurity, Key Laboratory of Jilin Province for Zoonoses Prevention and Control Academy of Military Medical Sciences Changchun China; ^5^ Division of HIV/AIDS and Sex‐transmitted Virus Vaccines National Institutes for Food and Drug Control Beijing China

**Keywords:** Ebola virus, therapeutic target, TM9SF2, viral entry, virus–host interactions

## Abstract

The high lethality of the Ebola virus (EBOV) underscores the critical need to understand its molecular lifecycle. Here, we systematically mapped EBOV‐host interactions using affinity purification‐mass spectrometry (AP‐MS) and proximity labeling, generating a comprehensive protein–protein interaction (PPI) network, of which 1728 are high‐confidence core PPIs involving nine EBOV proteins and 914 human interactors. Notably, we identified transmembrane 9 superfamily member 2 (TM9SF2) as a novel attachment factor that facilitates EBOV glycoprotein (GP)‐mediated entry. Mechanistic studies demonstrated that the receptor‐binding domain (RBD) of EBOV GP associates with the extracellular domain (ECD) of TM9SF2, enhancing cellular adhesion and viral attachment. Moreover, the anti‐TM9SF2 antibodies effectively attenuated EBOV GP‐mediated pseudovirus entry and infection in vitro. Additionally, *Tm9sf2* ^*+/*^ *^−^* knockout mice exhibited significantly reduced susceptibility to EBOV pseudoviruses. These findings provide critical insights into EBOV–host interactions and establish TM9SF2 as a promising therapeutic target for antiviral intervention.

## Highlights

- A comprehensive Ebola virus (EBOV)‐host protein–protein interaction (PPI) network consists of 1728 core high‐confidence interactions.
- TM9SF2 was identified as a crucial host attachment factor that facilitates EBOV pseudovirus infection, both in vitro and in vivo.
- Targeting TM9SF2 significantly impairs EBOV pseudovirus infection, offering a promising strategy for antiviral intervention.

## Graphical abstract

This study generates a comprehensive Ebola virus (EBOV)‐human protein–protein interactome, comprising 1728 core high‐confidence interactions. Further interactome analysis revealed the potential association of EBOV glycoprotein (GP) with the host factor TM9SF2. Subsequent mechanistic investigations confirmed that TM9SF2 functions as an attachment factor that, through its association with EBOV GP, promotes viral attachment and internalization to facilitate infection. These findings position TM9SF2 as a promising antiviral target.

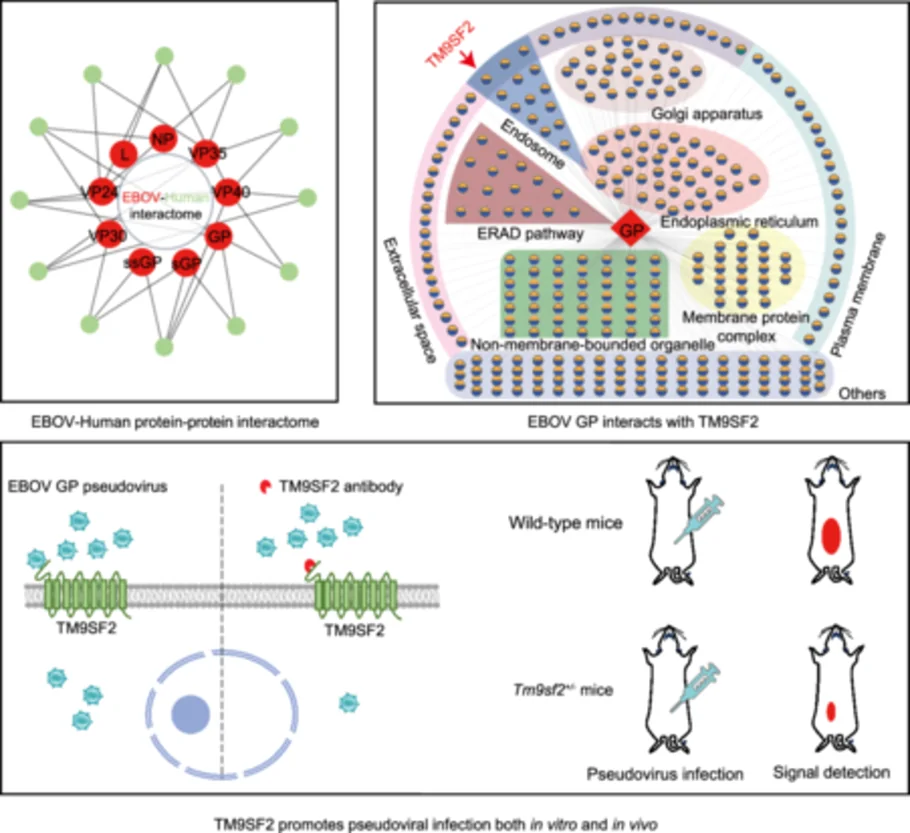

## 1 INTRODUCTION

Since its discovery in 1976, Ebola virus, a causative agent of hemorrhagic fever with a high fatality rate (50%–90%) [1, 2, 3], has triggered recurrent outbreaks over the past five decades. The unprecedented West Africa epidemic of 2013–2016 and the 2018–2020 outbreaks in the Democratic Republic of Congo underscored its enduring threat to global public health and economic development [4, 5].

EBOV is a single‐stranded, negative‐sense RNA virus with a genome of approximately 19 kb, containing seven genes that produce nine viral proteins with distinct structural and functional roles. The nucleoprotein (NP) encapsidates the viral genome and is a key component of the ribonucleoprotein complex (RNP) [6]. Viral protein VP35 acts as a polymerase cofactor that facilitates viral replication and transcription, while also antagonizing host interferon responses [7, 8]. The matrix protein VP40 orchestrates virion assembly and budding [9]. The primary product of the GP gene is the soluble glycoprotein (sGP), RNA editing by the viral polymerase complex produces a proportion of transcripts encoding the transmembrane GP, which forms homotrimeric spikes on the viral envelope to mediate viral entry. A different editing event generates a second smaller soluble glycoprotein, ssGP [10, 11]. VP30 is a nucleocapsid‐associated protein involved in transcriptional regulation [12]. VP24 enhances viral infectivity by suppressing interferon signaling through inhibition of the Janus kinase/signal transducer and activator of the transcription (JAK/STAT) pathway [13]. The L protein, a multifunctional RNA‐dependent RNA polymerase, catalyzes viral genome replication and transcription [14].

Interactions between EBOV proteins and host cellular factors are fundamental to the viral life cycle. However, the precise molecular mechanisms by which EBOV hijacks host pathways and perturbs cellular homeostasis remain poorly understood. A recent study using affinity purification‐mass spectrometry (AP‐MS) to map the EBOV–host interactome identified the ubiquitin ligase RBBP6 as a restriction factor that inhibits viral replication, underscoring the therapeutic potential of host‐targeted interventions [15]. However, this study did not include three EBOV proteins (sGP, ssGP and L), but also, like other AP‐MS‐based approaches, may miss transient or weak PPIs due to the inherent technical limitations of the method [16, 17]. To address these challenges, proximity labeling techniques based on engineered biotin ligases have been increasingly applied in virus‐host interactome mapping studies, enabling the capture of both stable and transient interactions and offering deeper insight into viral manipulation of host processes [18, 19, 20, 21].

In this study, we employed a combined AP‐MS and proximity labeling strategy to systematically map the interactions between EBOV proteins and human host factors. This approach yielded a comprehensive EBOV–human interactome comprising 6869 interactions, including a high‐confidence core dataset of 1728 PPIs involving all nine EBOV proteins and 914 host interactors, many of which are associated with pathways disrupted during infection. Among the novel interactions identified, we found that EBOV GP physically interacts with the transmembrane protein TM9SF2, via its receptor‐binding domain (RBD), which engages the extracellular domain (ECD) of TM9SF2 to facilitate viral attachment. Functional studies demonstrated that disrupting TM9SF2 expression significantly impairs GP‐mediated pseudovirus infection both in vitro and in vivo. These findings establish TM9SF2 as a previously unrecognized attachment factor involved in EBOV entry and a potential target for antiviral intervention.

## 2 RESULTS

### Construction of a comprehensive EBOV–human interactome network

To systematically investigate the interactions between EBOV proteins and host factors, we constructed an EBOV–human interactome using two complementary approaches: Flag/Strep tag‐based AP‐MS and proximity labeling. For the AP‐MS experiments, each of the nine EBOV proteins (Figure 1A) was individually expressed in HEK293T cells with N‐terminal 2 × Strep‐3 × Flag tags (Figure 1B), and protein complexes were purified in biological triplicates. In parallel, for proximity labeling, the EBOV proteins were fused at N‐terminus to the promiscuous biotin ligase TurboID. The subcellular localization of TurboID‐tagged viral proteins remained consistent with their native distribution, as confirmed by colocalization with Flag‐tagged viral proteins and biotinylated proximal proteins (Figures S1, S2). Biotinylated proteins were enriched using anti‐biotin antibody in triplicates (Figure 1C), and analyzed by liquid chromatography‐tandem mass spectrometry (LC‐MS/MS) (Figure 1D).

**Figure 1:**
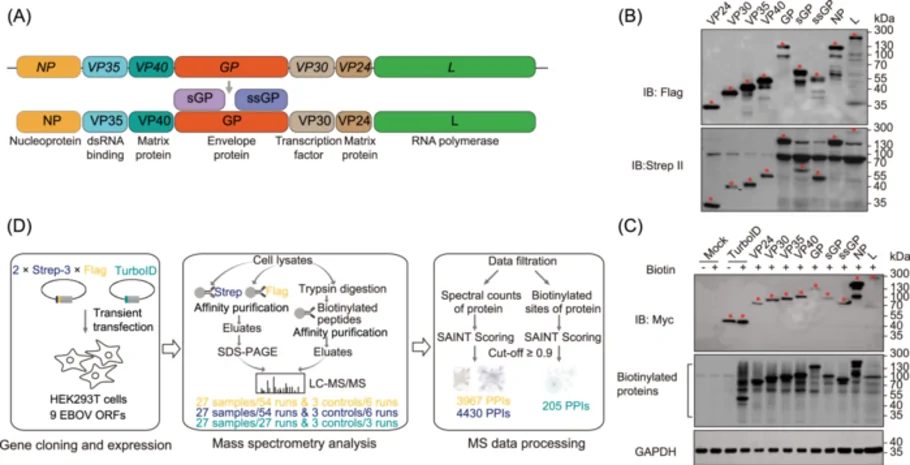
Screening strategies used for constructing the EBOV–human interactome. (A) Schematic representation of the EBOV genome and encoded viral proteins. (B) Western blot analysis confirming the expression of nine EBOV proteins fused with N‐terminal 2 × Strep‐3 × Flag tags in HEK293T cells, detected using anti‐Flag and anti‐StrepII antibodies. (C) Expression of EBOV proteins fused with TurboID was verified using anti‐Myc antibody, while biotinylated proteins were detected using Streptavidin‐HRP after biotin incubation. Target EBOV proteins are indicated by red asterisks. (D) Overview of the combined AP‐MS and proximity labeling (TurboID) strategies used to identify high‐confidence interactions. For AP‐MS, two LC‐MS/MS runs were analyzed per sample, comprising both Flag and Strep purifications, along with vector controls. For TurboID‐based proximity labeling samples, one LC‐MS/MS run was performed per sample, including respective TurboID‐only controls.

Both strategies yielded highly reproducible datasets, with an average Pearson correlation coefficient greater than 0.6 among biological triplicates (Figure S3A–C). Hierarchical clustering of the interactome heatmap revealed distinct interaction profiles for individual viral proteins (Figure S3D–F), indicating strong internal consistency and screening specificity. To refine the dataset, we implemented a stringent filtering pipeline to eliminate non‐specific interactors (Figure 2A), resulting in a comprehensive EBOV–human interactome comprising 6869 interactions between the nine EBOV proteins and 2643 human proteins (Figure 2B,C). Quantitative comparison between Flag and Strep AP‐MS revealed strong reproducibility (Spearman *r* = 0.824, Figure S3G), despite method‐specific differences arising from distinct negative controls. Method combination sensitivity analysis confirmed the ≥ 2 independent replicates criterion as an optimal balance, avoiding both the noise of single replicates and the over‐restriction of requiring TurboID (Figure S3H). BFDR threshold analysis further validated the high statistical stringency of all datasets (Figure S3I). Based on these observations, we further defined a high‐confidence core interactome as the subset of interactions supported by at least two out of three parallel pull‐down experiments (Flag AP‑MS, Strep AP‑MS—treated as independent replicates, not orthogonal strategies—and TurboID), yielding 1728 PPIs involving 914 host proteins (Figure 2C, Figure S4, and Tables S1–6).

**Figure 2:**
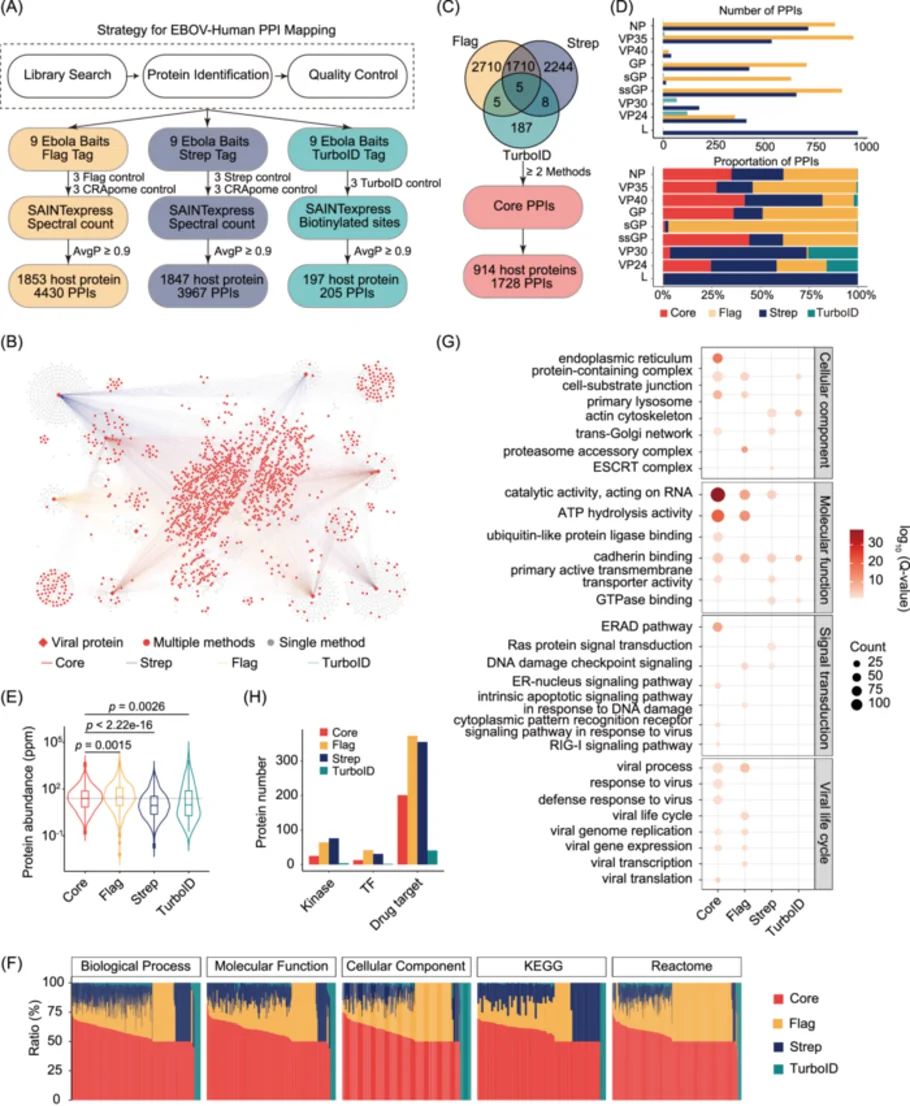
Bioinformatics analysis of the EBOV–human interactome. (A) Workflow for constructing a high‐confidence EBOV–human PPI network from multiple screening strategies. (B) Global landscape of EBOV–host interactions identified via AP‐MS, TurboID, and core integration. (C) Overlap of the host interactors across three datasets. Core PPIs are defined as interactions identified by at least two methods. (D) Number (upper panel) and proportion (lower panel) of host interactors associated with each EBOV protein across datasets. (E) Comparison of host protein abundance distributions among the AP‐MS, TurboID, and core datasets. Protein abundance is expressed in parts per million (ppm), representing the number of protein molecules per million total protein molecules. Unpaired Wilcoxon rank‐sum test was used to compare protein abundances between different methods. (F) Enrichment ratio of functional terms from GO, KEGG, and Reactome analyses. Enriched terms are represented as bars with indicated colors. (G) Gene Ontology enrichment analysis interactors from each dataset. (H) The protein numbers of kinases, transcription factors (TF), and drug targets among the AP‐MS, TurboID, and core datasets.

Our EBOV–host interactome recapitulated approximately half (80/170) of the interactions reported in a previous study [15], and overlapped with 91 of 354 PPIs listed in a curated EBOV–human interaction database [22] (Figure S5A). Additionally, we identified 17 of 71 host proteins previously found to be differentially expressed in plasma from EBOV survivors versus fatalities [23], revealing potential subnetworks relevant to early EBOV diagnosis (Figure S5B,C). Importantly, our screen recaptured 10 well‐characterized host factors implicated in EBOV infection, including interleukin enhancing factor 3 (ILF3) [24], the scaffolding protein IQ motif‐containing GTPase‐activating protein 1 (IQGAP1) [25], DNA topoisomerase I (TOP1) [26], tumor susceptibility gene 101 (TSG101) [27], vacuolar protein sorting 4 homolog A (VPS4A) [28], etc (Figure S5D). However, canonical EBOV host receptors such as Niemann‐Pick C1 (NPC1) and T‐cell immunoglobulin and mucin domain 1 (TIM‐1) were not identified as high‐confidence interactors in our dataset. This may be due to the context‐specific or transient nature of their interactions with viral GP, which are less amenable to capture under our experimental conditions.

### Analysis of the EBOV–host protein interaction network

We compared the PPI datasets generated by the three complementary methods and found them to be largely complementary (Figure 2C). The two AP‐MS datasets shared 1715 PPIs, whereas their overlap with the proximity labeling dataset was minimal, with only 10 and 13 shared interactions, respectively. Accordingly, the number of host interactors detected per viral bait varied markedly across methods (Figure 2D and Tables S1–6). The core interactome is heavily dominated by the AP‑MS datasets, with TurboID‑supported interactions accounting for only approximately 1.0% (18/1728) of the core set. This methodological bias reflects the inherent differences in capture efficiency for high‑versus low‑abundance interactors (Figure 2E) and underscores the value of integrating orthogonal approaches for a more complete interaction landscape.

These results indicate that no single method can fully capture the complexity of the EBOV‐host interactome landscape. For example, we constructed a subnetwork focused on the RHO GTPase effector pathway that previously implicated in EBOV infection [29] (Figure S6). Within this subnetwork, only 13.1% (24/183) of the host interactors were identified in more than one independent experiment. Notably, VP24 and VP30 interactors were more frequently detected via proximity labeling, whereas GP, L, NP, and VP35 primarily associated with proteins detected by AP‐MS.

To investigate the biological relevance of these interactions, we performed Gene Ontology (GO) and pathway enrichment analyses on the datasets derived from each method (Tables S7–11). The results showed the core interactome accounted for more than 50% of the significantly enriched terms, while the TurboID‐derived dataset contributed distinct functional insights not captured by AP‐MS alone (Figure 2F,G). Enrichment analyses of interactors for each viral protein revealed clear functional specialization (Figure S7, Tables S12–16). For instance, the interactors of GP, sGP, ssGP, VP24, and VP40 were significantly enriched in cellular components associated with membrane trafficking, including early/late endosome, cytoplasmic vesicle lumen, and transport vesicles—consistent with their known roles in viral entry and intracellular trafficking. More specifically, the interactors of both sGP and ssGP showed marked enrichment in vesicular trafficking, endosomal/lysosomal and secretory pathways, which aligns with the localization of these two soluble proteins in the secretory pathway. In contrast, interactors of L, NP, VP35, and VP30 were enriched in RNA polymerase and RNase complex, in line with their functions in viral replication and transcription [6, 30]. Although EBOV replicates in the cytoplasm, we identified 40 host proteins annotated as chromosome‐associated, primarily interacting with ssGP, NP, and VP30. These proteins may exert regulatory functions outside the nucleus, such as modulating RNA processing, nuclear‐cytoplasmic transport, or host stress responses. Moreover, several immune‐related and antiviral pathways, such as NF‐κB [31] and the ISG15 antiviral mechanism [32], were significantly enriched across datasets. These findings underscore the necessity of integrating multiple screening approaches to establish a robust and comprehensive EBOV–host interactome.

To identify potential therapeutic targets, we next focused on host protein kinases, transcription factors (TFs), and drug targets within our dataset. Kinases and TFs are key regulatory molecules that are frequently hijacked by viruses during infection. We identified 64, 76, and 4 kinases using the Flag, Strep, and TurboID datasets (Figure 2H, Table S17), respectively, with 25 found in the core interactome (detected by at least two independent experiments). Similarly, we identified 42, 31, and 2 TFs in the Flag, Strep, and TurboID datasets, respectively, with 13 forming core PPIs.

Given the relevance of drug repurposing in antiviral drug development, we examined known drug targets within our dataset [33, 34]. A total of 373, 355, and 41 potential drug targets were identified using the Flag, Strep, and TurboID‐tagged approaches, respectively, with 201 represented in the core interactome (Figure 2H, Table S17). Of these, at least 10 targets have been previously implicated in EBOV replication. For example, NP is known to recruit CAD (carbamoyl‐phosphate synthetase 2, aspartate transcarbamylase, and dihydroorotase) protein to viral inclusion bodies via its GLN domain to support pyrimidine biosynthesis required for EBOV genome replication and transcription [35]. While we did not detect a direct interaction between NP and CAD in our dataset, we observed interactions between CAD and several other viral proteins, including GP, sGP, ssGP, VP24 and VP30, suggesting that CAD may participate in multiple stages of the EBOV life cycle.

We also uncovered previously unreported interactions between NP and two host factors: phosphatidylserine synthase 1 (PTDSS1) and heme oxygenase‐1 (HMOX1). Knockdown of *PTDSS1* has been shown to significantly suppress EBOV production [36], while upregulation of HMOX1 attenuated viral replication and transcription [37]. Additionally, we found that the molecular chaperone heat‐shock‐protein family A (Hsp70) member 5 (HSPA5) interacts with both GP and ssGP, and its inhibition substantially impaired EBOV replication in vitro and in vivo [38]. These proteins represent promising candidates for antiviral drug development and warrant further investigation into their roles in EBOV pathogenesis.

### TM9SF2 facilitates EBOV GP‐mediated infection

To investigate the mechanisms of EBOV entry and identify potential host factors involved in viral invasion, we classified the core GP interactor dataset based on cellular component annotations. This analysis revealed that 304 core interactors are localized to membrane‐associated organelles, including endoplasmic reticulum, Golgi apparatus, and endosomes (Figure 3).

**Figure 3:**
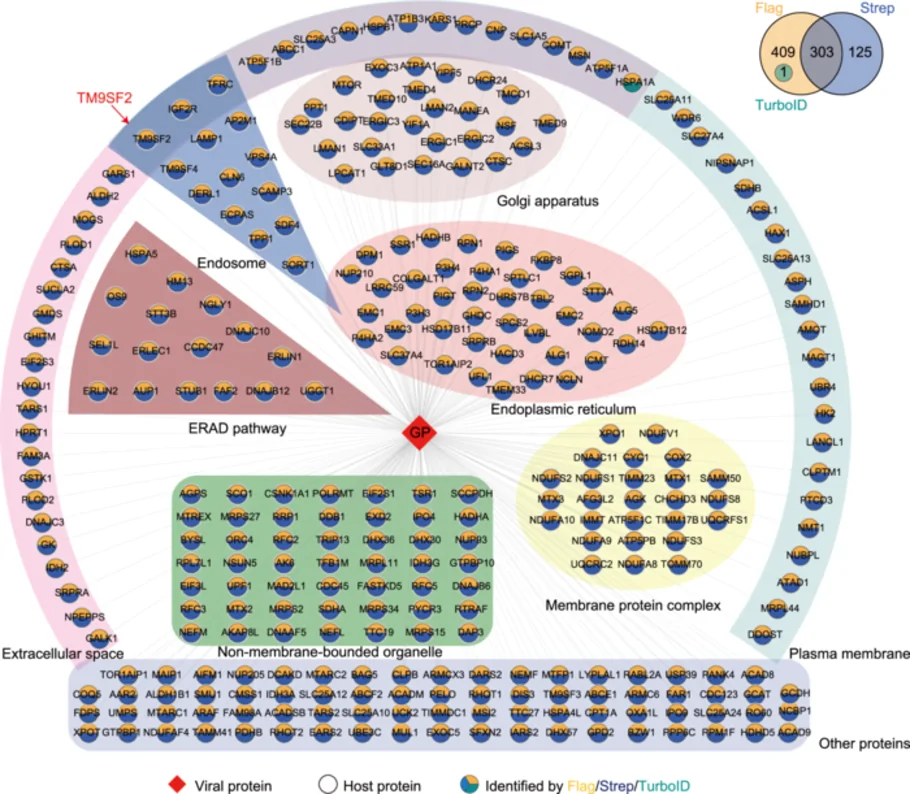
Core interaction network of EBOV glycoprotein (GP). A network view centered on EBOV GP (diamond node), with host proteins shown as circles color‐coded by detection method. Edges represent virus–host interactions. Host proteins sharing functional GO annotations are grouped by colored background blocks.

Through literature mining, we found that TM9SF2, a transmembrane protein localized to plasma membrane, has been implicated in multiple omics studies and associated with infections by various viruses, including adeno‐associated virus [39], vaccinia virus (VACV) [40], chikungunya virus (CHIKV) [41], influenza virus (IV) [42] and human immunodeficiency virus (HIV) [43]. These findings suggest a potential role for TM9SF2 in viral entry. However, its precise function in viral infections remains unclear, and its involvement in EBOV infection has not been previously established. As expected, co‐immunoprecipitation assays confirmed that TM9SF2 interacts with GP (Figure 4A).

**Figure 4:**
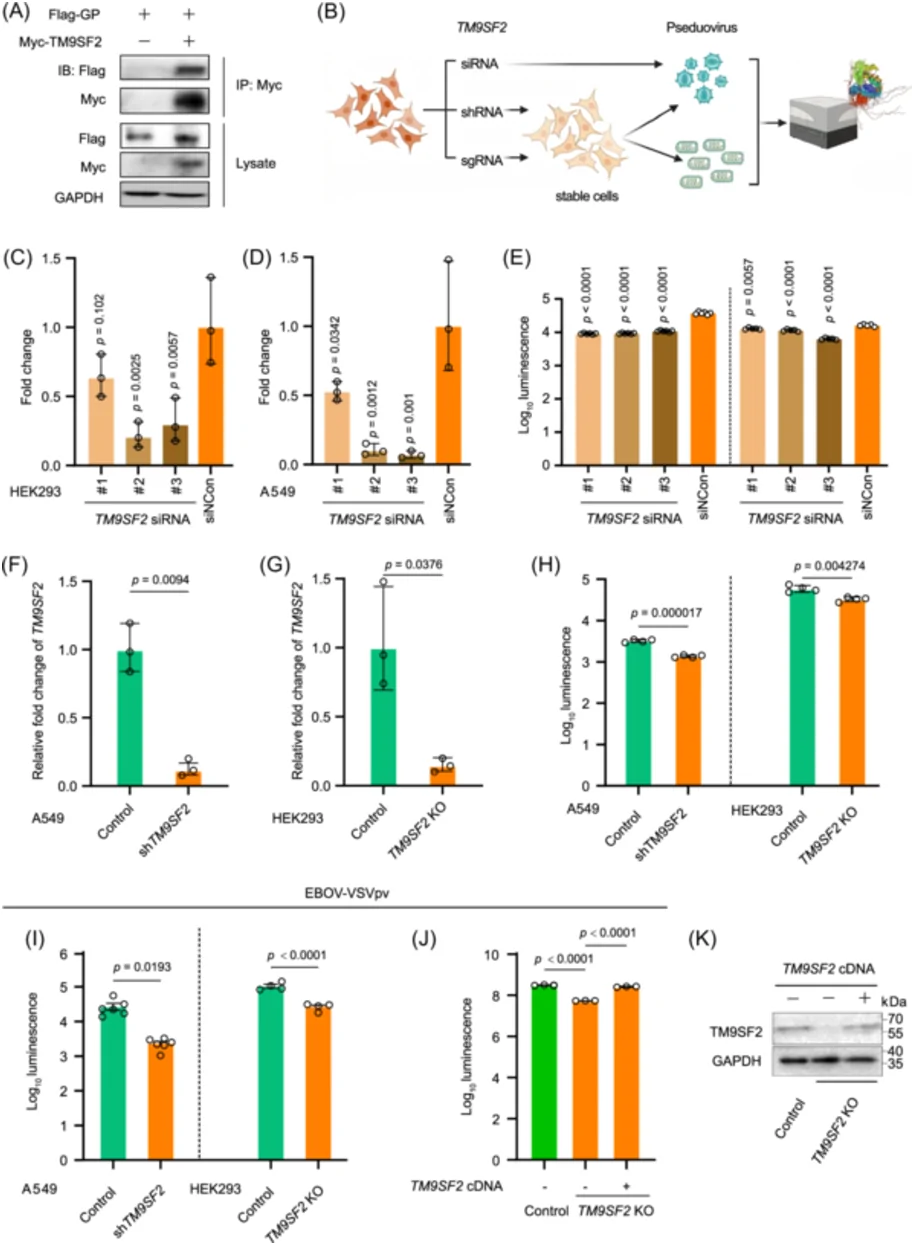
Suppression of TM9SF2 expression impairs EBOV GP‐mediated viral infection. (A) Co‐immunoprecipitation analysis of the interaction between EBOV GP and TM9SF2. HEK293T cells were co‐transfected with plasmids encoding GP and TM9SF2, followed by immunoprecipitation using an anti‐Myc antibody and immunoblotting with anti‐Myc or anti‐Flag antibodies, GAPDH was used as control. (B) Schematic overview of the experimental workflow used to suppress TM9SF2 expression and assess its role in EBOV pseudovirus infection. (C–E) HEK293 and A549 cells transfected with *TM9SF2*‐targeting siRNAs were infected with EBOV‐HIVpv. *TM9SF2* mRNA levels were analyzed by RT‐qPCR (C, D), and viral infectivity was quantified by measuring luminescence intensity (*n* = 5) (E). Each *TM9SF2* siRNA was compared with siNCon individually. (F) Quantitative analysis of *TM9SF2* mRNA expression in stable *TM9SF2* KD A549 and control cells. (G) Quantitative analysis of *TM9SF2* mRNA expression in *TM9SF2* KO HEK293 and control cells. (H) EBOV‐HIVpv infectivity was measured via luciferase activity in *TM9SF2* KD A549 cells, *TM9SF2* KO HEK293 cells, and their respective control cells (*n* = 4). (I) EBOV‐VSVpv infectivity was measured via luciferase activity in *TM9SF2* KD A549 cells (*n* = 6), *TM9SF2* KO HEK293 cells (*n* = 4), and their respective control cells. (J, K) Infectivity of EBOV‐VSVpv in control and *TM9SF2* KO HEK293 cells, with or without *TM9SF2* reconstitution (K) was assessed by luciferase activity (*n* = 3) (J). Statistical significance was assessed by one‐way ANOVA with Holm–Sidak post‐hoc test (C–E, J) or two‐tailed Student's *t*‐test (F–I).

To assess whether TM9SF2 contributes to EBOV GP‐mediated entry, we transfected HEK293 and A549 cells with short interfering RNAs (siRNAs) targeting *TM9SF2* and subsequently challenged them with EBOV GP‐pseudotyped non‐replicating viruses carrying a firefly luciferase (*Fluc*) reporter (EBOV‐HIVpv or EBOV‐VSVpv) (Figure 4B, Figure S8A). *TM9SF2* knockdown (KD) significantly impaired pseudovirus infection in both cell lines (Figure 4C–E).

To further validate these observations, we generated *TM9SF2‐*deficient cell lines using both shRNA‐mediated knockdown and CRISPR‐Cas9 knockout (KO) strategies. Successful *TM9SF2* KD in A549 cells (sh*TM9SF2*) and KO in HEK293 cells (*TM9SF2* KO) were confirmed (Figure 4F,G, and Figure S8B). As a positive control, we used the *NPC1* KO HEK293 cells, known to be resistant to EBOV entry [44] (Figure S8C,D). Consistent with the siRNA results, both KD and KO of *TM9SF2* significantly reduced EBOV GP‐mediated non‐replicating pseudovirus infection (Figure 4H,I). Conversely, ectopic expression of TM9SF2 in KO cells restored their susceptibility to EBOV‐VSVpv infection (Figure 4J,K).

To explore whether TM9SF2 plays a broader role in filovirus infection, we employed a non‐replicating VSV‐based pseudotyping system to generate MARV GP pseudotyped‐VSV particles (MARV‐VSVpv). Similarly to the results with EBOV pseudoviruses, loss of *TM9SF2* markedly reduced MARV‐VSVpv infection (Figure S8E). Collectively, these findings demonstrate that TM9SF2 is a critical host cofactor for filovirus entry mediated by viral glycoproteins.

### TM9SF2 is a host factor for replicative EBOV GP‐pseudotyped VSV

Given the observed requirement for TM9SF2 in EBOV GP‐mediated pseudovirus infection, we hypothesized that TM9SF2 may function during the early stages of viral entry, potentially at the level of attachment. To test this, we established a replication‐competent pseudotyping system by incorporating EBOV GP into a recombinant vesicular stomatitis virus (rVSV) backbone encoding a firefly luciferase reporter. rVSV‐Ebola particles were generated in BHK‐21 cells using T7 promoter‐driven expression of the VSV N, P, G and L proteins (Figure 5A). Western blot analysis confirmed efficient incorporation of EBOV GP into concentrated viral particles (Figure 5B). Viral titration and time‐course infection assays revealed that luciferase activity positively correlated with both viral input and infection duration, confirming efficient GP‐mediated entry and replication (Figure 5C,D).

**Figure 5:**
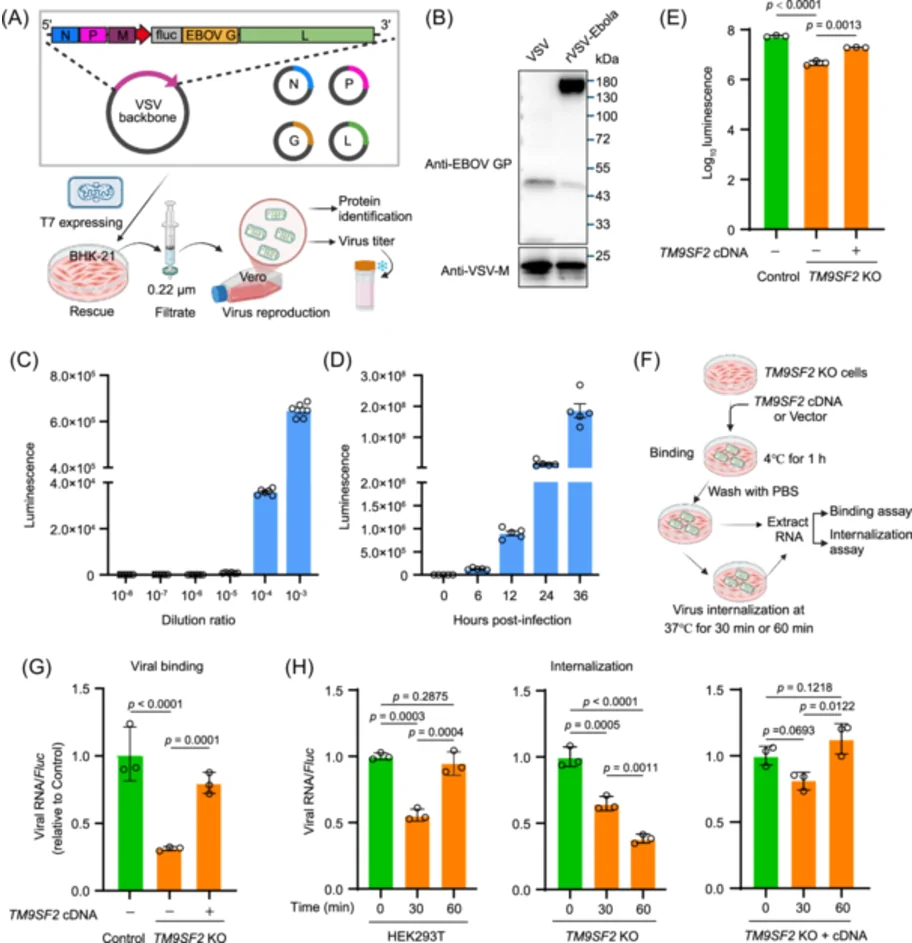
TM9SF2 functions as a host factor for EBOV GP‐mediated infection. (A) Schematic representation of the generation of replication‐competent VSV pseudotyped with EBOV GP (rVSV‐Ebola). (B) Immunoblot analysis of purified rVSV‐Ebola particles using antibodies against EBOV GP and VSV matrix protein (M), with wild‐type VSV as a control. (C) Viral titers of rVSV‐Ebola were determined by infecting Vero cells with 10‐fold serial dilutions (10^−3^ to 10^−8^). Luciferase activity was measured 24 h post‐infection to assess infectivity. (D) Replication kinetics of rVSV‐Ebola in Vero cells. Cells were infected with rVSV‐Ebola, and luciferase activity was measured at indicated time points. (E) HEK293 control and *TM9SF2* KO cells, with or without TM9SF2 reconstitution, were infected with rVSV‐Ebola (IFU = 1). Viral infectivity was quantified by luciferase activity. (F) Schematic diagram of the experimental design for assessing viral attachment and internalization of rVSV‐Ebola. (G) Assessment of viral attachment. HEK293 control and *TM9SF2* KO cells, with or without TM9SF2 reconstitution, were incubated with rVSV‐Ebola (IFU = 1) at 4°C for 60 min. Cell‐associated viral RNA was extracted and quantified by qPCR. (H) Assessment of viral internalization. Following 60‐min attachment at 4°C, which served as the 0‐min baseline control, cells were then shifted to 37°C for 30 or 60 min to permit viral internalization. Intracellular viral RNA was extracted and quantified by qPCR. Data are presented as means ± SD (*n* = 3). Statistical significance was determined using one‐way ANOVA followed by Holm–sidak post‐hoc test.

Infection assays in *TM9SF2* KO cells demonstrated a marked reduction in rVSV‐Ebola infectivity (Figure 5E), consistent with our findings using non‐replicative pseudoviruses. Importantly, re‐expression of TM9SF2 in KO cells restored susceptibility to infection, further supporting its role as a functional proviral factor.

To dissect the stage at which TM9SF2 facilitates EBOV GP‐mediated entry, we quantified viral binding and internalization by measuring cell‐associated firefly luciferase mRNA following infection (Figure 5F). *TM9SF2* KO cells displayed significantly reduced levels of both surface‐bound and internalized pseudovirus particles compared to control cells. Restoration of TM9SF2 expression in KO cells substantially increased pseudovirus binding and internalization (Figure 5G,H), indicating that TM9SF2 promotes early entry events. Together, these data identify TM9SF2 as a previously unrecognized host factor that facilitates EBOV GP‐mediated infection, particularly by enhancing viral binding and internalization during the initial stages of the viral life cycle.

### TM9SF2 interacts with the receptor‐binding domain of EBOV GP to facilitate viral attachment

To elucidate the molecular basis of the interaction between TM9SF2 and GP, we first characterized the domain architecture and structural organization of both proteins. GP is cleaved into GP1 and GP2 subunits, which form a trimeric spike complex, with GP1 capping the membrane fusion‐active GP2 [45]. TM9SF2 is a multi‐pass transmembrane protein consisting of an N‐terminal signal peptide, an extracellular/luminal domain (ECD), and nine transmembrane domains [46] (Figure S9A). Given the extracellular orientation of TM9SF2_ECD_ and the surface exposure of GP1, we hypothesized a potential interaction between these regions during the early stages of viral attachment. GST pull‐down assays confirmed a physical interaction between TM9SF2 and GP (Figure 6A), consistent with co‐immunoprecipitation data (Figure 4A). Subcellular localization analysis showed predominant localization of TM9SF2 at the plasma membrane, confirmed by co‐staining with the lipophilic dye DiI (Figure 6B), in line with previous reports [47]. Moreover, TM9SF2 exhibited marked co‐localization with GP, supporting the hypothesis of a membrane‐associated interaction during early viral entry.

**Figure 6:**
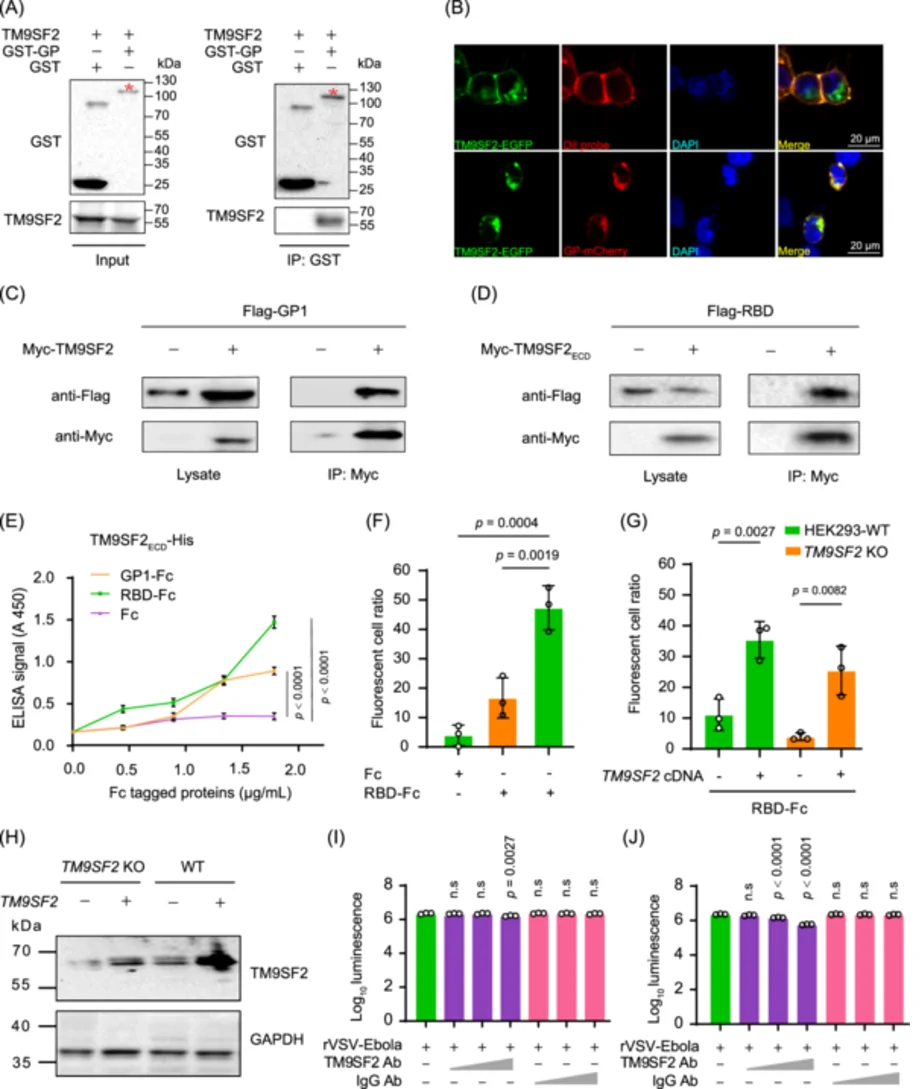
TM9SF2‐EBOV GP interaction mediates pseudovirus infection. (A) GST pull‐down assay showing the interaction of TM9SF2 with GP. Asterisks indicate GST‐GP protein. (B) Colocalization of TM9SF2 with GP. HEK293 cells expressing TM9SF2‐EGFP were stained with membrane probe Dil or co‐transfected with GP‐mCherry. Nuclei were counterstained with DAPI. (C) Co‐immunoprecipitation analysis of the interaction between TM9SF2 and EBOV GP1. (D) Co‐immunoprecipitation analysis of the interaction between TM9SF2_ECD_ and GP‐RBD. (E) ELISA‐based binding assay demonstrates direct interaction of TM9SF2_ECD_‐His with GP1‐Fc and RBD‐Fc. (F, G) Binding efficiency of RBD‐Fc to wild‐type or *TM9SF2* KO HEK293 cells, with or without TM9SF2 reconstitution. PE‐conjugated anti‐Fc antibody to detect RBD‐Fc binding to cells via flow cytometry. Data are presented as means ± SD (*n* = 3). (H) Western blot analysis confirming TM9SF2 overexpression in wild‐type and *TM9SF2* KO HEK293 cells. GAPDH served as the loading control. (I, J) Inhibition of rVSV‐Ebola (IFU = 1) entry by TM9SF2 antibodies at the indicated concentrations (6, 30, and 150 μg/mL). HEK293 cells were pre‐incubated with TM9SF2 antibodies (Thermo Fisher, I; Abcam, J) or control IgG, followed by rVSV‐Ebola infection. Viral infectivity was assessed by luciferase activity. Statistical significance was determined using two‐way ANOVA followed by Holm–Sidak post‐hoc test.

To investigate the specific region of GP involved in TM9SF2 binding, co‐immunoprecipitation (co‐IP) assays were performed using the GP1 subunit, revealing a robust interaction with TM9SF2 (Figure 6C). Since the receptor‐binding domain (RBD, 54–201 aa) of GP1 is essential for host receptor engagement [48], we hypothesized that TM9SF2 may directly contact this region. Co‐expression of the TM9SF2 extracellular domain (TM9SF2_ECD_, 1–300 aa) with GP1‐RBD in HEK293T cells, followed by immunoprecipitation, revealed a direct interaction between TM9SF2_ECD_ and GP1‐RBD (Figure 6D).

To quantitatively assess this interaction, we purified recombinant His‐tagged TM9SF2_ECD_, Fc‐tagged GP1 and RBD proteins (Figure S9B). ELISA‐based binding assays demonstrated that both GP1‐Fc and RBD‐Fc bound TM9SF2_ECD_ in a dose‐dependent manner, with significant binding observed at concentrations above 1.5 μg/mL. In contrast, control Fc fragments exhibited minimal nonspecific binding (Figure 6E). We next assessed the functional consequences of this interaction in a cell‐based adhesion assay (Figure S9C). Flow cytometry analysis revealed that RBD binding was reduced approximately 66% in *TM9SF2* KO cells compared to wild‐type controls, and this defect was rescued by TM9SF2 re‐expression (Figure 6F–H, Figures S10, S11).

To further validate the involvement of TM9SF2 in viral entry, we tested whether blocking TM9SF2 with specific antibodies could inhibit infection. Two commercial TM9SF2 antibodies significantly inhibited rVSV‐Ebola infection in a dose‐dependent manner, with notable inhibition observed at 150 μg/mL (Figure 6I,J). However, ELISA‐based assays revealed that these antibodies did not block the TM9SF2‐GP1 interaction *in vitro* (Figure S9D), potentially due to epitope masking when TM9SF2 is immobilized on plates. These findings suggest that TM9SF2‐targeting antibodies inhibit infection by interfering with GP attachment at the cell surface.

Collectively, these data establish TM9SF2 as a previously unrecognized cell‐surface interactor for EBOV GP. Mechanistically, its extracellular domain associates with the RBD of GP1, likely through interactions with partially accessible or conformationally flexible surfaces on virion‐associated GP during the early attachment step. This interaction promotes efficient viral binding and internalization, highlighting TM9SF2 as a potential therapeutic target for blocking EBOV infection.

### Murine TM9SF2 facilitates EBOV pseudovirus infection in vivo

To further assess the role of TM9SF2 in EBOV infection under physiological conditions, we generate *Tm9sf2* ^+/^^−^ mice on a C57BL/6 background using CRISPR/Cas9‐mediated genome editing. Specifically, sgRNAs and homologous oligonucleotides were designed to target exons 6–8 of the *Tm9sf2* gene (Figure S12A). Mutant mice carrying the *Tm9sf2* ^+/^^−^ allele were identified by PCR genotyping and confirmed by DNA sequencing. As intended, this strategy produced a precise 3534 bp deletion encompassing exons 6–8, resulting in a residual 619 bp fragment (Figure S12B). PCR amplification and DNA sequencing analysis validated the successful deletion of the targeted genomic region in the *Tm9sf2* ^+/^^−^ mice (Figure S12C,D), confirming the establishment of a heterozygous knockout model.

To determine whether TM9SF2 affects susceptibility to EBOV pseudovirus infection *in vivo*, *Tm9sf2* ^+/^^−^ and WT mice of both sexes were challenged intraperitoneally with 1000 IFU of EBOV‐HIVpv. Following infection, we monitored bioluminescent signals daily until signal attenuation was observed. *Tm9sf2* ^+/^^−^ male mice exhibited markedly reduced bioluminescent intensity throughout the infection period (Figure 7A, left), with significant reductions on day 8 compared to WT controls (Figure 7B). Remarkably, *Tm9sf2* ^+/^^−^ female mice showed an even more pronounced suppression of infection (Figure 7A, right), displaying significantly luminescent signals than their WT counterparts at all monitored time points (Figure 7C).

**Figure 7:**
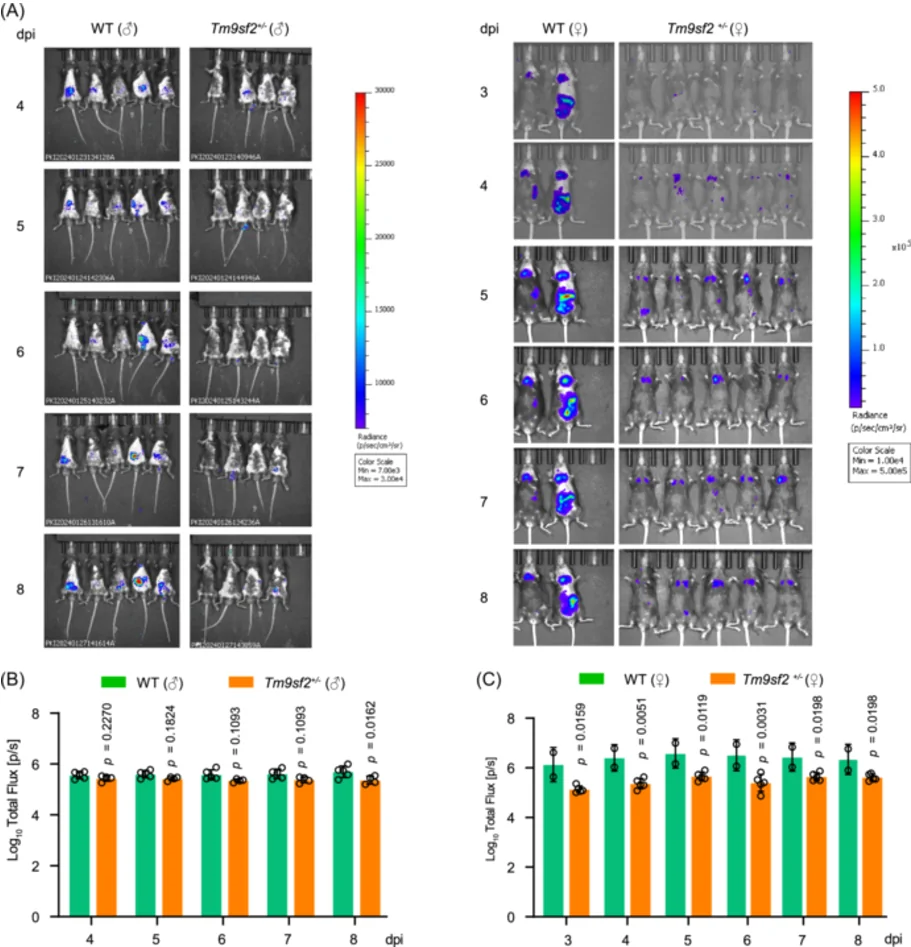
TM9SF2 promotes pseudoviral infection in vivo. (A) Wild‐type and *Tm9sf2* ^+/^^−^ mice were intraperitoneally infected with EBOV‐HIVpv. Whole‐body bioluminescence was monitored daily post‐infection. (B, C) Quantification of bioluminescent signals in EBOV‐HIVpv‐infected mice at the indicated time points. Statistical significance was determined using two‐way ANOVA followed by Holm–Sidak post‐hoc test.

These findings demonstrate that *Tm9sf2* haploinsufficiency substantially impairs EBOV‐GP‐mediated infection. Together with our in vitro results, these data identify TM9SF2 as a critical host attachment factor for efficient EBOV GP‐mediated pseudovirus entry and propagation in vivo, underscoring its potential as a therapeutic target for EBOV infection.

## 3 DISCUSSION

In this study, we systematically mapped a global PPI network between Ebola virus and human proteins. By integrating two complementary screening approaches, AP‐MS and proximity labeling, we identified 6869 interactions involving nine EBOV proteins and 2643 unique human host factors, including a high‐confidence core dataset of 1728 interactions.

Our comparative analysis revealed that the nature of EBOV–human interactome is highly influenced by the experimental platform used. AP‐MS preferentially captured high‐abundance and transient interactors, whereas proximity labeling effectively identified low‐abundance partners. These complementary strengths highlight the necessity of combining orthogonal approaches to construct a more complete and nuanced view of viral–host interactions. The resulting dataset provides a valuable resource for dissecting the molecular basis of EBOV pathogenesis.

A recent study identified 447 host proteins associated with EBOV proteins using BioID2‐based proximity labeling [49], of which only 10 interactions overlapped with this study. Such limited overlap is consistent with known variability in proximity labeling studies and likely reflects differences in biotin ligases properties (BioID2 vs. TurboID), tagging positions, labeling durations, and criteria for defining high‐confidence interactors. Despite these discrepancies, the datasets are complementary and together offer a more comprehensive picture of EBOV–host interaction networks.

Among the identified host factors, we focused on TM9SF2, a membrane‐associated interactor of the EBOV glycoprotein. Functional studies demonstrated that inhibition or genetic disruption of TM9SF2 markedly attenuates EBOV pseudovirus infection, establishing TM9SF2 as a host factor required for efficient viral entry. TM9SF2 belongs to the transmembrane 9 superfamily (TM9SF1–4), characterized by nine transmembrane domains and previously implicated in small molecule transport, endosomal trafficking, and glycosphingolipid homeostasis via localization to the early endosomes and Golgi apparatus [41, 50]. Previous studies have associated TM9SF2 with pathogen interactions, including phagocytosis and bacterial internalization in *Drosophila* [51]. Additionally, TM9SF2 has emerged in several large‐scale screens as a candidate factor involved in viral infection [40, 52, 53]. Moreover, TM9SF2 is constitutively expressed in multiple cell types (e.g., macrophages, dendritic cells and endothelial cells) and tissues (e.g., liver, spleen and lung) that are major targets of EBOV. Subcellularly, it localizes to the plasma membrane and endosomes, consistent with its role in protein trafficking. This broad expression pattern aligns with the broad tissue tropism of EBOV and supports TM9SF2 as a physiologically relevant attachment factor modulating viral entry.

Our mechanistic analyses revealed that the extracellular domain of TM9SF2 (TM9SF2_ECD_) binds with the RBD of EBOV GP1. Immunofluorescence assays confirmed co‐localization TM9SF2 and GP at the plasma membrane; the physical proximity of TM9SF2 and GP at the plasma membrane places TM9SF2 at the site of initial virus–host contact, supporting the hypothesis that TM9SF2 plays a role in the early stages of viral entry. Functional validation assays showed that TM9SF2 acts as a host attachment factor. Viral adhesion of rVSV‐Ebola pseudovirus was significantly impaired *TM9SF2* KO cells and rescued by TM9SF2 re‐expression. Interestingly, although TM9SF2‐targeting antibodies failed to block the TM9SF2_ECD_–GP1 interaction in vitro (likely due to epitope masking in the ELISA setup), they effectively suppressed pseudovirus infection in a dose‐dependent manner, suggesting that antibody‐mediated disruption of TM9SF2 function can impair viral entry at cellular level.

To contextualize the role of TM9SF2 in the broader EBOV entry process, we compared its function with that of NPC1, an essential endosomal receptor required for membrane fusion at a later stage of infection. In contrast, TM9SF2 appears to function during the early phase of viral attachment at the plasma membrane, acting as an attachment or entry cofactor that enhances early entry steps. This distinction may explain why the antiviral effect of TM9SF2 disruption is moderate compared to *NPC1* knockout. Moreover, other redundant attachment factors such as TIM‐1 and DC‐SIGNR may partially compensate for the loss of TM9SF2. Nevertheless, the modest rescue efficiency observed in our assays represents a limitation of the current study. Our data therefore indicate that TM9SF2 contributes to, but is not solely responsible for, EBOV GP‐mediated entry. Although our functional experiments focused on EBOV GP, it remains unclear whether TM9SF2‑mediated viral attachment is specific to EBOV, conserved across other ebolaviruses, or extends more broadly to non‑filoviruses. The observation that TM9SF2 promotes MARV pseudovirus entry suggests it may serve as a filovirus‑wide attachment factor; however, direct testing using glycoproteins from additional ebolaviruses as well as representative non‑filoviruses will be required to clarify the scope of this interaction. In vivo, *Tm9sf2* ^+/^^−^ mice exhibited significantly reduced susceptibility to EBOV‐HIVpv infection compared to WT controls, further supporting TM9SF2's role as a critical host factor for EBOV entry under physiological conditions.

Despite these findings, several limitations of this study should be acknowledged. First, this study was mainly performed in HEK293 and A549 cells, which do not fully recapitulate the physiological cell types targeted by Ebola virus. While these models facilitated mechanistic studies, further validation in more relevant cells such as THP‑1 or Huh7 will be needed to confirm the relevance of TM9SF2 in EBOV infection. Second, due to the classification of EBOV as a biosafety level 4 (BSL‐4) pathogen, our functional validation relied on HIV‐ or VSV‐based pseudovirus systems, which served as reliable and widely accepted surrogates for studying EBOV entry under lower biosafety conditions. Nonetheless, validation using authentic EBOV under BSL‐4 containment remains essential and will be pursued in future studies when access becomes available. Moreover, although our study demonstrated that TM9SF2 promotes EBOV GP‐mediated entry using multiple approaches, critical controls, including glycoprotein‐deficient pseudoviruses or unrelated viral glycoproteins were not included. Such controls are necessary to exclude nonspecific effects, particularly given that TM9SF2 has been linked to multiple viral infections. Third, although our study identified a broad spectrum of EBOV‐interacting host proteins, we focused mechanistic investigation on TM9SF2 due to its membrane localization, functional tractability, and initial evidence supporting a role in viral entry. Further studies are warranted to explore the roles of other candidate host factors revealed in the screening. In addition, while several host factors, including TIM‐1, NPC1, and the newly identified TM9SF2, have been implicated in EBOV GP‐mediated entry, the dominant receptor(s) governing viral tissue and species tropism remain elusive. It is likely that EBOV utilizes a multistep, receptor‐cofactor‐based entry strategy, whereby distinct factors mediate sequential steps of attachment, internalization, trafficking, and membrane fusion. Understanding this layered mechanism may open new avenues for antiviral intervention. Although our data indicate that TM9SF2 promotes both viral attachment and internalization, the current study does not resolve whether TM9SF2 functions solely as an adhesion molecule or actively participates in endocytic uptake (e.g., via macropinocytosis) or subsequent endosomal escape. For instance, TM9SF2 and NPC1 act within the same sequential entry pathway but govern distinct spatiotemporal steps: TM9SF2 functions early to support viral attachment, internalization, and endocytic delivery of virions, whereas NPC1 mediates the final GP‐triggered membrane fusion event within late endosomes. High‑resolution time‑resolved colocalization studies with early endosome, late endosome, and lysosome markers will be required to determine whether TM9SF2 accompanies GP into the endolysosomal pathway. Finally, although complete *Tm9sf2* knockout in mice is embryonically lethal, heterozygous *Tm9sf2* ^+/−^ mice exhibited partial but interpretable protective phenotypes upon pseudovirus challenge, supporting the role of TM9SF2 as a proviral host factor. However, given the lack of littermate controls, the potential impact of batch differences on the observed genotype effects cannot be fully ruled out. Future investigations using littermate controls derived from heterozygous intercrosses may help to strengthen these conclusions. The more pronounced protection in female *Tm9sf*2^+/^^−^ mice suggests a potential sex‐specific role for TM9SF2 in EBOV entry. This observation, though its underlying mechanism remains unclear, highlights an important avenue for future research into TM9SF2 function across sexes, potentially involving sex hormones or behavior observation during EBOV infection. Further development of conditional knockout models or humanized mouse systems will be essential to clarify the physiological contribution of TM9SF2 to EBOV infection and disease progression. Notably, our in vivo studies employed non‐replicating EBOV HIV pseudoviruses to specifically and quantitatively assess GP‐mediated viral entry in *Tm9sf2* ^+/−^ mice, thereby eliminating confounding effects from viral replication and dissemination. Future studies using replication‐competent rVSV‐Ebola in vivo will be important to validate the role of TM9SF2 in viral propagation and pathogenesis.

Clinically, these findings have several important implications. First, TM9SF2 is a cell‐surface, multi‐spanning membrane protein with an extracellular domain that engages with the EBOV GP receptor‐binding domain, suggesting it may serve as a druggable interface for therapeutic targeting. Second, the involvement of TM9SF2 in multiple viral infections, as suggested by prior omics studies, raises the possibility that it represents a shared entry cofactor across diverse enveloped viruses. This highlights the potential for TM9SF2‐targeting strategies to be repurposed for broad‐spectrum antiviral applications. Furthermore, as host‐directed therapies may be less prone to resistance mutations compared to viral‐targeted drugs, TM9SF2 represents a promising candidate for the development of monoclonal antibodies or small‐molecule inhibitors aimed at blocking viral entry.

Taken together, our study identifies TM9SF2 as a novel cell‐surface attachment factor facilitating EBOV entry via engagement with GP, and underscores its potential as both a mechanistic entry cofactor and a therapeutic target. These findings expand our understanding of filovirus–host interactions and provide a foundation for future antiviral discovery efforts targeting host components of the viral life cycle.

## 4 CONCLUSION

In conclusion, this study provides a comprehensive analysis of the EBOV‐host PPI network, identifies TM9SF2 as a critical host factor in EBOV GP‐mediated pseudovirus entry by promoting viral attachment and internalization. This positions TM9SF2 as a promising therapeutic target and offers a potential strategy for antiviral intervention. These findings advance our understanding of EBOV entry mechanisms and pave the way for developing host‐targeted therapies against EBOV and other viral infections.

## 5 METHODS

### Gene cloning and plasmid construction

The coding sequences of all nine EBOV proteins from *Zaire* ebolavirus (GenBank accession number: KP759676) were synthesized with N‐terminal 2 × Strep‐3 × Flag epitope tags (GenScript) and cloned into the pVAX1 expression vector for AP‐MS screening. For proximity labeling experiments, the same EBOV coding sequences were subcloned into pcDNA3.1‐myc‐TurboID vector. Detailed information on the expression constructs and EBOV protein sequences is provided in Table S18. All plasmids are available from the corresponding author upon request.

### Immunofluorescence microscopy

HEK293T cells were seeded onto confocal microscopy dishes and transfected with the indicated constructs. At 24 h post‐transfection (cells intended for proximity labeling were incubated with 50 μM biotin for 10 min), cells were washed with PBS, fixed with 4% paraformaldehyde (PFA, sc‐281692, Santa Cruz) for 10 min, permeabilized on ice with 0.2% Triton X‐100 (sc‐29112, Santa Cruz, Dallas, TX) for 10 min, and blocked with 1% bovine serum albumin (BSA, w/v) at room temperature for 1 h. Primary antibodies were diluted in 1% BSA and applied for 1 h in the dark. After washing with PBS, cells were counterstained with DAPI (D1306, Invitrogen) and imaged using a Zeiss LSM880 confocal microscope (Carl Zeiss, Oberkochen) equipped with a 40× objective lens.

### Affinity purification of flag‐ and strep‐tagged proteins for mass spectrometry

To generate samples for mass spectrometric analysis, the experiment was conducted with three independent biological replicates. For each replicate, HEK293T cells (approximately 5 × 10^6^ per 10‐cm dish) were transfected with 7 μg pLVX‐2 × Strep‐3 × Flag EBOV expression plasmids using polyethyleneimine (PEI, 23966‐1, Polysciences) at a 5:1 PEI‐to‐DNA ratio (w/v). At 24 h post‐transfection, cells were harvested and lysed by RIPA buffer (50 mM Tris, pH 7.4; 150 mM NaCl; 1% Triton X‐100; 1% sodium deoxycholate; 0.1% SDS; supplemented with protease inhibitor cocktail (P8340, Sigma‐Aldrich)) on ice. Lysates were sonicated (4°C, 30 W, 4 s on/4 s off for 2 min) and clarified by centrifugation. Protein concentration was measured using the BCA assay (23227, Thermo Fisher Scientific). For Flag‐tag affinity purification, 1 mg cell lysate was incubated with 1 μg of anti‐Flag‐tag monoclonal antibody (M185‐3L, MBL) at 4°C for 6 h on a rotator, followed by incubation with 30 μL protein A/G agarose beads (sc‐2003, Santa Cruz) overnight. For Strep‐tag purification, 1 mg of lysates were incubated with 30 μL Strep‐Tactin Sepharose beads (6‐6350‐002, IBA Lifesciences, Göttingen, Germany) overnight at 4°C. Beads were washed three times with RIPA buffer and resuspended in 50 μL of RIPA and 50 μL of 2 × Laemmli sample buffer, followed by boiling at 95°C for 10 min. Eluates were resolved via SDS‐PAGE, stained with Coomassie Brilliant Blue (1610436, Bio‐Rad) for 30 min, and destained using commercial destaining solution (P0017A, Beyotime). The gel was excised and diced into ~1 mm^3^ pieces, and transferred to 1.5 mL microcentrifuge tubes. Gel pieces were destained by shaking at 1500 rpm in 500 μL destain solution (50% 50 mM triethylammonium bicarbonate, TEAB (T7408, Thermo Fisher Scientific, Waltham, MA) and 50% acetonitrile) until clear, dehydrated in acetonitrile, and dried by vacuum centrifugation. Disulfide bonds were reduced by incubating gel pieces in 100 μL of 20 mM DTT (dissolved in 50 mM TEAB) at 56°C for 45 min with shaking (1000 rpm), then alkylated in 100 μL of 30 mM IAA (in 50 mM TEAB) for 30 min at room temperature in the dark. Gel pieces were dehydrated with acetonitrile and dried by vacuum centrifugation. Trypsin digestion was carried out overnight at 37°C in TEAB buffer. Peptides were extracted using 50% acetonitrile with 0.1% TFA, combined with the supernatant, dried by vacuum centrifugation, and desalted using StageTips.

### TurboID‐based proximity labeling for mass spectrometry

TurboID‐based proximity labeling was performed as previously described [54]. To generate samples for mass spectrometric analysis with three independent biological replicates. For each replicate, we performed proximity labeling as follows: 24 h after transfection of TurboID‐tagged EBOV constructs, cells were incubated with 50 μM biotin (B4501, Sigma‐Aldrich) for 10 min at 37°C. Cells were then harvested and lysed in 8 M urea containing 50 mM TEAB, followed by sonication (4°C, 30 W, 4 s on/4 s off, for 2 min). Approximately 6 mg of total protein was prepared for triplicate biological analyses. For each replicate, 2 mg of protein was reduced with 20 mM DTT for 30 min at 56°C and transferred to Amicon Ultra‐0.5 Centrifugal Filter Unit (UFC5010BK, Millipore). Samples were washed with urea buffer (8 M urea, 100 mM Tris‐HCl pH 8.5), alkylated with 50 mM IAA for 30 min in the dark. and further washed with urea buffer followed by 50 mM TEAB. Trypsin digestion was performed overnight at 37°C. Digested peptides were washed, eluted with 50 mM TEAB, dried by vacuum centrifugation, and resuspended with 1 mL of IAP buffer (50 mM MOPS, pH 7.2; 10 mM sodium phosphate; 50 mM NaCl). Anti‐biotin antibody‐conjugated beads (ICP0615, ImmuneChem) were added and incubated overnight at 4°C with rotation. Beads were washed three times with PBS, and bound peptides were eluted twice with 50 mL of 0.15% trifluoroacetic acid (TFA, T6508, Sigma‐Aldrich). Elutes were dried by vacuum centrifugation and desalted using StageTips.

### Mass spectrometry

The extracted peptides were reconstituted in 8 μL solvent A (0.1% formic acid in water) and analyzed by using a Q‐Exactive HF‐X coupled to the Thermo UltiMat 3000 RSLCnano system (Thermo Fisher Scientific). Briefly, 6 µL of sample was loaded onto a silica‐based nano‐ESI column (360 µm OD × 150 µm ID) packed with a 15 cm bed of ReproSil‐Pur 120 C18‐AQ 1.9 µm particles (tip size: 3–5 µm). Peptide separation and analysis were then performed using an 88‐min LC‐MS/MS method. The mobile phases consisted of solvent A and solvent B (0.1% formic acid in 80% acetonitrile). Peptides were separated using the following gradient at a flow rate of 0.3 μL/min for the first 10 min and 0.6 μL/min thereafter: 0–10 min, 6% B; 10–15 min, 6–10% B; 15–70 min, 10–30% B; 70–80 min, 30–40% B; 80–80.1 min, 40–95% B; 80.1–85 min, 95% B; 85–85.1 min, 95–6% B; 85.1–88 min, 6% B.

Mass spectrometry data acquisition was performed with the following settings: full MS scans (MS1) were acquired over a range of m/z 350–1550 at a resolution of 120,000, with an automatic gain control (AGC) target of 3 × 10^6^ and a maximum iron time of 20 ms. For tandem MS (MS2), the top 25 most intense precursor ions from each MS1 scan were isolated using a 1.6 m/z window, fragmented with a normalized collision energy of 27, and analyzed over an m/z 200–2000 range at a resolution of 15,000, with an AGC target of 2 × 10^4^ (minimum 1 × 10^3^), an intensity threshold of 3.3 × 10^4^, and a maximum iron time of 30 ms; dynamic exclusion was set to 15 s, the first fixed mass was m/z 110, precursor selection was monoisotopic, and ions with charge states of unassigned, 1, 8, or >8 were excluded.

### Mass spectrometry data analysis

Raw mass spectrometry data were searched against the Uniprot Swiss‐Prot Human database (v201503), supplemented with the EBOV protein sequences, using Mascot software (v2.3.2). A decoy database generated by reversing all protein sequences was appended to the target database for accurate estimation of peptide probabilities and the false discovery rate (FDR), with the FDR threshold set at 0.01. Search parameters were set as follows: trypsin was selected as the proteolytic enzyme with up two missed cleavages allowed; peptide charges were restricted to 2+, 3+, and 4+; peptide error tolerance was 20 ppm; MS/MS error tolerance was 0.05 Da. Carbamidomethylation of cysteine (+57.02146 Da) was set as a fixed modification, while protein N‐terminal acetylation (+42.010565 Da), methionine oxidation (+15.995 Da), and cysteine mercaptoethanol modification (DeStreak of cysteine, +75.998285) were set as variable modifications. Peptide‐ and protein‐level false discovery rates were set to 0.01 using Pepdistiller (v.1.28) [55] and PANDA (v1.1.4) [56], respectively. An ion score cut‐off of 10 was applied for peptide identification confidence.

### Bioinformatic analysis

To construct a high‐confidence EBOV‐host interactome, all datasets were subjected to stringent quality control, including comparisons with three independent negative controls (empty Flag, Strep, and TurboID vectors). For AP‐MS datasets, interactions were scored using SAINTexpress (v3.6.3) [57] based on spectral counts with a confidence cut‐off of ≥0.9. To further reduce background, common contamination proteins listed in the CARPome [58] database were excluded. For proximity labeling datasets, interactions were scored by SAINTexpress based on the number of biotinylated sites, using the confidence threshold ≥0.9.

Gene Ontology and pathway enrichment analyses were performed using the R packages clusterProfiler (v4.4.4) [59] and ReactomePA (v1.42.0) [60]. Terms with a false discovery rate (Q‐value) <0.05 and at least two associated genes were considered significantly enriched. Redundant GO terms were removed using the R package GOSemSim (v2.18.1) [61] with Lin's semantic similarity metric and a medium similarity threshold of 0.7. Data visualizations were generated using multiple R packages: ggplot2 for dot plots and ternary plots, pheatmap for heatmaps, and corrplot for correlation matrices. mRNA expression data were obtained from the Human Protein Atlas (HPA) cell line data RNA dataset, while protein abundance data were retrieved from PaxDB database (v4.1) [62]. Interaction networks were visualized using Cytoscape (v3.7.1).

Transcription factors (TFs) were curated from AnimalTFDB (v4.0), a comprehensive repository of experimentally validated and predicted TFs across animal species. Drug‐target interaction data were extracted from DrugBank (v5.1.12), focusing on high‐confidence drug–protein relationships. Drug metadata and annotations were programmatically using the R package dbdataset, ensuring standardized data retrieval and reproducibility.

### Co‐immunoprecipitation and western blot analysis

HEK293T cells were transfected with the indicated plasmids and harvested 24 h post‐transfection. Cells were washed with PBS and lysed in co‐IP buffer (50 mM Tris‐HCl pH 7.5, 100 mM NaCl, 1.5 mM MgCl_2_, 0.2% (v/v) NP‐40, 5% (v/v) glycerol, protease inhibitor cocktail). Lysates were sonicated on ice (30 W, 4 s on/4 s off, for 2 min) and centrifuged at 12,000 *g* for 10 min at 4°C to remove debris. The supernatants were incubated with protein A/G magnetic beads (HY‐K0202, MedChemExpress, Monmouth Junction) that had been pre‐bound to anti‐Myc antibody (M192‐3MS, Medical & Biological Laboratories Co.) for 2 h at 4°C, and then incubated for 16 h at 4°C with rotation. Beads were subsequently washed three times with co‐IP buffer, and bound protein complexes were eluted in 50 μL co‐IP buffer supplemented with 50 μL 2 × Laemmli sample buffer. Whole cell lysates and immunoprecipitated proteins were analyzed by Western blot using the indicated antibodies. Signals were detected using chemiluminescence (ECL, 32209; Thermo Fisher Scientific).

### Cell culture and recombinant protein purification

HEK293 cells were maintained in high‐glucose Dulbecco's Modified Eagle Medium (DMEM, C0162‐811, CellWorld) supplemented with 10% (v/v) fetal bovine serum (FBS, 04‐001‐1A, Biological Industries, Kibbutz Beit Haemek), penicillin‐streptomycin (30‐002‐CI, Corning), and cultured at 37°C in a humidified incubator with 5% CO_2_. For recombinant protein expression, HEK293 cells were transfected with expression plasmids encoding TM9SF2_ECD_‐His, GP1‐Fc, or RBD‐Fc. His‐tagged proteins were purified using Ni‐NTA agarose resin (CW0894S, CoWin Biosciences), and Fc‐tagged proteins were purified using protein G agarose (10600‐P07E‐RN, Sino Biological) according to the manufacturers' protocols. Purified proteins were validated by SDS‐PAGE and Western blot using anti‐His or anti‐Fc antibodies.

### Generation of *TM9SF2* KO cell lines

CRISPR/Cas9‐mediated knockout of *TM9SF2* was performed using the lentiCRISPRv2 system [63]. Two sgRNAs targeting exon 1 of the human *TM9SF2* gene (sgRNA1: 5'–CGCCGGAGCGGCGCTTTCTA–3' and sgRNA2: 5'–GAGCGGCGCTTTCTACCTGC–3' were designed via CRISPOR. These sgRNAs exhibited CDF specificity scores of 97 and 96, respectively, and their off‐target analyses are documented in Table S19. The sgRNAs were subsequently cloned into the lentiCRISPRv2 vector. Lentiviral particles were produced by co‐transfecting HEK293T cells (5 × 10^6^ cells per 10‐cm dish) with a total of 8 μg plasmids consisting of lentiCRISPRv2‐sg*TM9SF2*, pMD2.G and psPAX2 at a 5:2:3 mass ratio using PEI as the transfection reagent. At 48 h post‐transfection, viral supernatants were collected and centrifuged to remove cell debris. HEK293 cells seeded in six‐well plate were transduced with the viral supernatants. After 48 h, the transduced cells were selected with 1 μg/mL puromycin (P8833, Sigma‐Aldrich) for 5–7 days to establish a mixed *TM9SF2* knockout population. To obtain genetically homogeneous cell lines, single‑cell clones were subsequently isolated from this population by limiting dilution and expanded to establish the final *TM9SF2* KO cells. Successful gene disruption was confirmed by genomic PCR and Western blot.

### RNAi assay

Transient knockdown of *TM9SF2* was performed using siRNAs (targeting and non‐targeting control) purchased from RiboBio. Transfections were carried out using Lipofectamine™ RNAiMAX (13778100, Thermo Fisher Scientific) according to the manufacturer's protocol. Sequences of the siRNAs are provided in Table S20. Knockdown efficiency was assessed by Western blot or RT‐qPCR.

shRNAs targeting human *TM9SF2* gene or a scrambled control (based on the pLVRU6H vector) were obtained from GeneCopoeia. Sequences of shRNAs are shown in Table S20. For lentivirus production, HEK293T cells were co‐transfected with shRNA‐expressing plasmids along with packaging vectors psPAX2 and pMD2.G using Lipofectamine™ 3000 (L3000008, Thermo Fisher Scientific). At 48 h post‐transfection, culture supernatants containing lentiviral particles were collected, clarified by centrifugation, and used to infect A549 cells. Transduced cells were selected with 300 μg/mL Hygromycin B (ant‐hg‐1, InvivoGen) for stable knockdown.

### Construction of the replication‐incompetent EBOV GP‐pseudotyped viruses

Replication‐defective lentiviral particles pseudotyped with EBOV GP were generated as previously described [64]. Briefly, HEK293T/17 cells were co‐transfected with pLentiCMV‐Luc2, pCMV‐dR8.2‐dvpr, and expression plasmids encoding either EBOV GP or MARV GP, using PEIpro transfection reagent (101000017, Polyplus Transfection). At 48 h post‐transfection, culture supernatants were harvested, clarified by low‐speed centrifugation (1000 × *g* for 10 min), and filtered through a 0.45 μm syringe filter to remove cell debris. Viral titers were determined using the Lenti‐X p24 Rapid Titer Kit (632200, Takara‐bio). For infection assays, pesudoviruses were used to infect HEK293 cells. At 48 h post‐infection, luciferase activity was measured using the Bright‐Lumi™ Firefly Luciferase Assay Kit (RG052S, Beyotime) according to the manufacturer's instructions.

To generate replication‐incompetent VSV pseudoviruses bearing EBOV or MARV glycoproteins (designated EBOV‐VSVpv or MARV‐VSVpv), HEK293T/17 cells were transfected with the plasmids encoding EBOV GP (Zaire strain) or MARV GP using Lipofectamine 3000 (L3000008, Invitrogen), respectively. Concurrently, cells were infected with VSVΔG*G (1:1000 dilution), a VSV vector lacking the glycoprotein gene (G) but pseudotyped with VSV‐G and harboring a luciferase reporter. After 6 h, supernatants were aspirated, and cells were washed three times with PBS containing 2% FBS. Complete DMEM (10% FBS) was added, and pseudoviruses were harvested from the supernatant at 24 h post‐infection. Virions were filtered through a 0.45 μm membrane and stored at −80°C until use.

### Construction of a recombinant VSV antigenome encoding EBOV GP

To generate VSV particles pseudotyped with EBOV GP, we constructed a full‐length cDNA clone of the VSV antigenome in which the native VSV‐G gene was replaced with the GP gene from the Zaire ebolavirus strain. Additionally, a firefly luciferase reporter gene was inserted as an independent transcriptional unit upstream of the GP gene to facilitate quantitative monitoring of infection.

### Generation of replication‐competent VSV‐Ebola pseudovirus (rVSV‐Ebola)

Replication‐competent VSV‐based pseudovirus was rescued using a plasmid‐based system as previously described [65]. Briefly, BHK‐21 cells were infected with vaccinia virus vTF7‐3 (expressing T7 RNA polymerase) for 2 h. After removal of the viral inoculum, cells were co‐transfected with the VSV antigenome plasmid and helper plasmids (encoding VSV N, P, G, and L proteins under the control of the T7 promoter) using Lipofectamine™ 3000. At 48 h of post‐transfection, culture supernatants were harvested, filtered through a 0.22 μm membrane, and subsequently passaged onto Vero cells to amplify the rescued virus. The virus was serially passaged three to four times at 2–3 day intervals to achieve higher titers. Viral RNA was extracted, and the inserted GP gene was amplified by RT‐qPCR and confirmed by DNA sequencing. Virus stocks were aliquoted and stored at −80°C for downstream applications.

### GST pulldown assay

To assess direct interaction between TM9SF2 and EBOV GP, the GP coding sequence was cloned into the pGEX‐4T‐2 vector to express a GST‐tagged fusion protein. The construct was transformed into *E. coli* BL21 cells, cultured at 37°C for 2 h, and induced with 0.5 mM isopropyl β‐d‐thiogalactopyranoside (IPTG, I6758, Sigma‐Aldrich) for 4 h at 37°C. Bacterial cells were harvested, lysed in bacterial lysis buffer, and sonicated. After centrifugation, the soluble protein fraction was incubated with Glutathione Sepharose beads (17‐0756‐05, Cytiva) at 4°C for 2 h to immobilize GST‐GP. Beads were washed three times and subsequently incubated overnight with lysates from HEK293T cells expressing TM9SF2. After three additional washes, bound proteins eluted and analyzed by SDS–PAGE followed by Western blot using the indicated antibodies.

### TM9SF2 capture ELISA

High‐binding 96‐well ELISA plates (3590, Corning) were coated with purified His‐tagged TM9SF2 protein (1 μg/mL in PBS) and blocked with PBS containing 3% bovine serum albumin (PBSA). Recombinant GP1‐Fc or RBD‐Fc proteins (0.5–2 μg/mL in PBSA) were added to the wells and incubated for 1 h at 37°C. After extensive washing to remove unbound proteins, HRP‐conjugated goat anti‐human IgG (ZB‐2304, ZSGB‐BIO, Beijing, China) was added and incubated for 40 min at 37°C. The plates were washed thoroughly, and Fc‐tagged proteins were detected with a TMB substrate (P0209, Beyotime).

### Cell adhesion assay

HEK293 cells were harvested, washed, and incubated with purified RBD‐Fc proteins (10 μg/mL) at 4°C for 1.5 h to allow binding. Unbound proteins were removed by washing, and cells were subsequently incubated with PE‐conjugated goat anti‐human IgG (A56015‐PE, Thermo Fisher Scientific) for 1 h at 4°C. Cells were washed, fixed by 4% paraformaldehyde, and analyzed flow cytometry to assess Fc‐tagged protein binding. For each group, we analyzed approximately 10^4^ cells and quantified the percentage of PE‐positive cells, which reflects the proportion of cells bound by RBD‐Fc, Fc fragment alone served as a negative control.

### Viral attachment and internalization assays

For the viral attachment assay, cells were washed with cold PBS and incubated with rVSV‐Ebola (input focus units, IFU = 100) at 4°C for 1 h to permit viral binding without internalization. After incubation, unbound pseudovirus was removed by two washes with cold PBS, and total RNA was extracted. Viral RNA (vRNA) levels were quantified by RT‐qPCR. For the internalization assay, after the initial 1 h binding at 4°C, cells were shifted to 37°C and incubated for 30 or 60 min to allow viral entry. Cells were then washed, total RNA was extracted, and internalized vRNA was quantified by RT‐qPCR.

### Real‐time quantitative reverse‐transcription PCR (RT‐qPCR)

Total RNA from cells was isolated using TRIzol (15596026, Sigma‐Aldrich) according to the manufacturer's instructions. The total RNA was then reverse transcribed into cDNA by using ReverTra Ace™ qPCR RT Master Mix (FSQ‐201, TOYOBO, Osaka, Japan). PCR amplification was carried out with Taq Pro Universal SYBR qPCR Master Mix (Q712‐02, Vazyme). The PCR cycling conditions used were 95°C for 5 min, followed by 40 cycles of 95°C for 10 s, 60°C for 20 s. Single peaks in the melting curve analysis indicated the specificity of the amplicons. RT‐qPCR primers of *TM9SF2*, *GAPDH*, and *Fluc* are listed in Table S20. Relative mRNA expression levels of the selected genes were calculated using the ΔCt method, with *GAPDH* mRNA serving as the internal control.

### Animals, in vivo infection, and bioluminescence imaging

All animal experiments were approved by the Institutional Animal Care and Use Committee of the National Institute for Food and Drug Control (NIFDC) with the approved number of NIFDC (F) 2024(B)043. Wild‐type C57BL/6N mice were obtained from Charles River Laboratories, Wilmington, MA. *Tm9sf2* knockout mice (stock No. S‐KO‐12586) were generated on a C57BL/6N background and are maintained by Cyagen Biosciences. Genomic DNA was extracted from mouse tail tips using Quick Genotyping Assay Kit (D0065M, Beyotime), and genotypes were confirmed by PCR following the manufacturer's instructions. PCR primer sequences used for *Tm9sf2* deletion genotyping are listed in Table S20. All animals were maintained under specific pathogen‐free conditions, with a maximum of five mice per cage, in a controlled environment (20°C, 50 ± 10% relative humidity). Six to eight‐week‐old *Tm9sf2* ^+/^^−^ and wild‐type (WT) mice were utilized in this study. A total of 10 *Tm9sf2* ^+/^^−^ mice (5 females, 5 males) and 10 WT mice (5 females, 5 males) were divided into groups based on sex. Mice were randomly allocated into groups according to body weight via an online random number generator to minimize systematic bias. Mice were intraperitoneally inoculated with 1000 IFU of EBOV‐HIV pseudoviruses; this dose was chosen based on previous studies employing the same pseudovirus system in murine models, as it generates stable and detectable bioluminescence signals without inducing severe animal distress or mortality, consistent with our non‐pathogenic readout design. All procedures, including pseudoviruses inoculation, in vivo bioluminescence imaging, data acquisition, and quantification, were conducted by researchers blinded to group assignments, and the blinding was only lifted after data analysis was completed. In vivo bioluminescence imaging was executed at predetermined time points post‐infection to monitor viral dissemination, and luminescence signals were quantified using Living Image software with standardized region of interest settings applied across all samples to reduce measurement variability, accompanied by repeated measurements to ensure data robustness. Strict humane endpoints were established in accordance with institutional animal care guidelines, including over 20% body weight loss, pronounced lethargy, and impaired mobility, and mice meeting these criteria were humanely euthanized immediately. All mice were housed under identical environmental and feeding conditions to further mitigate the influence of genetic background and environmental factors. All experiments were replicated independently three times, and representative data are shown.

### Statistical analysis

Unless otherwise specified, all experiments were biological independently repeated at least three times. Statistical analyses were conducted using GraphPad Prism 9.5.1 (GraphPad Software). Data are presented as the mean ± standard deviation (SD). Statistical significance was determined using two‐tailed Student's *t*‐tests (*p < 0.05; **p < 0.01; ***p < 0.001; ****p < 0.0001; n.s, not significant). For multiple comparations, statistical significance was determined using one‐way ANOVA followed by Holm–Sidak post‐hoc test. The normality of data distribution was assessed using the Shapiro–Wilk test, and homogeneity of variances was verified by Levene's test. All groups exhibited a normal distribution of residuals and equal variances (*p* > 0.05), satisfying the prerequisites for parametric one‑way ANOVA.

## AUTHOR CONTRIBUTIONS

**Limin Shang**: Writing—original draft; methodology; funding acquisition; investigation; visualization. **Shouwen Du**: Investigation; writing–original draft; supervision; formal analysis; funding acquisition. **Yuchen Liu**: Software; data curation; writing—original draft; formal analysis. **Letian Li**: Validation; investigation. **Xi Wu**: Investigation; formal analysis. **Yanzhi Yuan**: Methodology; investigation. **Chaozhi Jin:** Software; investigation. **Qingkun Mi**: Methodology; investigation. **Yuhang Wang**: Visualization; investigation. **Xiaoshuang Shi**: Visualization; investigation. **Jiamin Wang**: Formal analysis; validation. **Guoqing Zhang**: Investigation; software. **Yidan Ma**: Investigation; validation. **Weijin Huang**: Resources; supervision. **Jigang Wang**: Writing—review & editing; resources. **Jian Wang**: Writing—review & editing; conceptualization; funding acquisition; supervision; project administration. **Chang Li**: Writing—review & editing; conceptualization; funding acquisition; project administration; resources.

## CONFLICT OF INTEREST STATEMENT

The authors declare no conflicts of interest.

## ETHICS STATEMENT

This study was approved by the Institutional Animal Care and Use Committee of the National Institute for Food and Drug Control (NIFDC) (approval number NIFDC (F) 2024(B)043) and conducted in compliance with all applicable institutional and national guidelines for animal care and use.

## Supporting information


**Figure S1:** Confocal imaging of EBOV proteins with or without TurboID tags.
**Figure S2:** Proximity labeling of EBOV proteins in HEK293T cells.
**Figure S3:** Correlation analysis of interactome results using different screening strategies.
**Figure S4:** Core EBOV‐host PPI network.
**Figure S5:** Comparison of EBOV–host interactome with published dataset.
**Figure S6:** Subnetwork of Reactome pathway “RHO GTPase Effectors.”
**Figure S7:** Functional enrichment analysis of host interactors for each EBOV protein.
**Figure S8:** Generation of *TM9SF2* and *NPC1* knockout cells for EBOV and MARV pseudovirus infection assays.
**Figure S9:** TM9SF2 promotes EBOV infection via adhesion.
**Figure S10:** Flow cytometry analysis of RBD‐Fc binding to wild‐type and *TM9SF2* KO HEK293 cells.
**Figure S11:** TM9SF2‐dependent binding of RBD‐Fc in WT, KO, and rescued HEK293 cells.
**Figure S12:** Construction and genotyping of *Tm9sf2* ^+/^^−^ mice.

**Table S1:** High confidence protein–protein interactions identified by Strep‐tag AP‐MS.
**Table S2:** High confidence protein–protein interactions identified by Flag‐tag AP‐MS.
**Table S3:** High confidence protein–protein interactions identified by TurboID proximity labeling.
**Table S4:** Core protein–protein interactions identified by multiple independent experiments.
**Table S5:** List of negative controls used in SAINT Express analysis.
**Table S6:** Contribution of TurboID‐supported interactions within the core EBOV–human interactome, broken down by viral bait.
**Table S7:** Enriched Gene Ontology biological process.
**Table S8:** Enriched Gene Ontology molecular function (GO:MF) terms identified by different methods.
**Table S9:** Enriched Gene Ontology cellular component (GO:CC) terms identified by different methods.
**Table S10:** Enriched KEGG pathways identified by different methods.
**Table S11:** Enriched Reactome pathways identified by different methods.
**Table S12:** Enriched Gene Ontology biological process (GO:BP) terms for each bait.
**Table S13:** Enriched Gene Ontology molecular function (GO:MF) terms for each bait.
**Table S14:** Enriched Gene Ontology cellular component (GO:CC) terms for each bait.
**Table S15:** Enriched KEGG pathway terms for each bait.
**Table S16:** Enriched Reactome pathway terms for each bait.
**Table S17:** List of identified transcription factors, kinases, and known drug targets in each dataset.
**Table S18:** List of EBOV proteins used in interaction screening assays.
**Table S19:** sgRNA off‐target assessment.
**Table S20:** List of oligonucleotides used in this study.

## Data Availability

The raw mass spectrometry data of this study have been deposited in the iProX repository and can be accessed at https://www.iprox.cn//page/SCV017.html?query=PXD078530. The data used for analyzing the article's charts and graphs has been uploaded to GitHub (https://github.com/data168/iMetaOmics1322141). Supplementary materials (figures, tables, graphical abstract, slides, videos, Chinese translated version, and updated materials) may be found in the online DOI or iMeta Science http://www.imeta.science/imetaomics/. The data that support the findings of this study are openly available in iProx at https://www.iprox.cn//page/SCV017.html?query=PXD078530.

## References

[1] Report of an International, Commission . 1978. “Ebola Haemorrhagic Fever in Zaire, 1976.” Bull World Health Organ 56: 271–293 https://www.ncbi.nlm.nih.gov/pubmed/307456 PMC2395567 307456

[2] Report of a, W. H. O. International Study Team . 1978. “Ebola Haemorrhagic Fever in Sudan, 1976. Report of a WHO/International Study Team.” Bull World Health Organ 56: 247–270 https://www.ncbi.nlm.nih.gov/pubmed/307455 PMC2395561 307455

[3] Khan, Ali S. , F. Kweteminga Tshioko , David L. Heymann , Bernard Le Guenno , Pierre Nabeth , Barbara Kerstiëns , Yon Fleerackers , et al. 1999. “The Reemergence of Ebola Hemorrhagic Fever, Democratic Republic of the Congo, 1995.” The Journal of Infectious Diseases 179(Suppl 1): S76–S86. 10.1086/514306 9988168

[4] Jacob, Shevin T. , Ian Crozier , William A. Fischer , Angela Hewlett , Colleen S. Kraft , Marc‐Antoine de La Vega , Moses J. Soka , et al. 2020. “Ebola Virus Disease.” Nature Reviews Disease Primers 6: 13. 10.1038/s41572-020-0147-3 PMC7223853 32080199

[5] Feldmann, Heinz , Armand Sprecher , and Thomas W. Geisbert . 2020. “Ebola.” New England Journal of Medicine 382: 1832–1842. 10.1056/NEJMra1901594 32441897

[6] Jain, Sahil , Ekaterina Martynova , Albert Rizvanov , Svetlana Khaiboullina , and Manoj Baranwal . 2021. “Structural and Functional Aspects of Ebola Virus Proteins.” Pathogens 10: 1330. 10.3390/pathogens10101330 PMC8538763 34684279

[7] van Tol, Sarah , Birte Kalveram , Philipp A. Ilinykh , Adam Ronk , Kai Huang , Leopoldo Aguilera‐Aguirre , Preeti Bharaj , et al. 2022. “Ubiquitination of Ebola Virus VP35 at Lysine 309 Regulates Viral Transcription and Assembly.” PLoS Pathogens 18: e1010532. 10.1371/journal.ppat.1010532 PMC9119628 35533195

[8] Basler, Christopher F . 2015. “Innate Immune Evasion by Filoviruses.” Virology 479–480: 122–130. 10.1016/j.virol.2015.03.030 PMC4424075 25843618

[9] Gordon, Tamsin B. , Joshua A. Hayward , Glenn A. Marsh , Michelle L. Baker , and Gilda Tachedjian . 2019. “Host and Viral Proteins Modulating Ebola and Marburg Virus Egress.” Viruses 11: 25. 10.3390/v11010025 PMC6357148 30609802

[10] Baseler, Laura , Daniel S. Chertow , Karl M. Johnson , Heinz Feldmann , and David M. Morens . 2017. “The Pathogenesis of Ebola Virus Disease.” Annual Review of Pathology: Mechanisms of Disease 12: 387–418. 10.1146/annurev-pathol-052016-100506 27959626

[11] Sakurai, Yasuteru . 2015. “Ebola Virus Host Cell Entry.” Uirusu 65: 71–82. 10.2222/jsv.65.71 26923960

[12] Biedenkopf, Nadine , Julia Schlereth , Arnold Grünweller , Stephan Becker , and Roland K. Hartmann . 2016. “RNA Binding of Ebola Virus VP30 Is Essential for Activating Viral Transcription.” Journal of Virology 90: 7481–7496. 10.1128/JVI.00271-16 PMC4984652 27279615

[13] Fanunza, Elisa , Aldo Frau , Marco Sgarbanti , Roberto Orsatti , Angela Corona , and Enzo Tramontano . 2018. “Development and Validation of a Novel Dual Luciferase Reporter Gene Assay to Quantify Ebola Virus VP24 Inhibition of IFN Signaling.” Viruses 10: 98. 10.3390/v10020098 PMC5850405 29495311

[14] Yuan, Bin , Qi Peng , Jinlong Cheng , Min Wang , Jin Zhong , Jianxun Qi , George F. Gao , and Yi Shi . 2022. “Structure of the Ebola Virus Polymerase Complex.” Nature 610: 394–401. 10.1038/s41586-022-05271-2 PMC9517992 36171293

[15] Batra, Jyoti , Judd F. Hultquist , Dandan Liu , Olena Shtanko , John Von Dollen , Laura Satkamp , Gwendolyn M. Jang , et al. 2018. “Protein Interaction Mapping Identifies RBBP6 as a Negative Regulator of Ebola Virus Replication.” Cell 175: 1917–1930.e13. 10.1016/j.cell.2018.08.044 PMC6366944 30550789

[16] Morwitzer, M Jane , Sarah R. Tritsch , Lisa H. Cazares , Michael D. Ward , Jonathan E. Nuss , Sina Bavari , and St Patrick Reid . 2019. “Identification of RUVBL1 and RUVBL2 as Novel Cellular Interactors of the Ebola Virus Nucleoprotein.” Viruses 11: 372. 10.3390/v11040372 PMC6521077 31018511

[17] Batra, Jyoti , Hiroyuki Mori , Gabriel I. Small , Manu Anantpadma , Olena Shtanko , Nawneet Mishra , Mengru Zhang , et al. 2021. “Non‐Canonical Proline‐Tyrosine Interactions with Multiple Host Proteins Regulate Ebola Virus Infection.” The EMBO Journal 40: e105658. 10.15252/embj.2020105658 PMC8441301 34260076

[18] Liu, Xiaonan , Sini Huuskonen , Tuomo Laitinen , Taras Redchuk , Mariia Bogacheva , Kari Salokas , Ina Pöhner , et al. 2021. “SARS‐CoV‐2‐Host Proteome Interactions for Antiviral Drug Discovery.” Molecular Systems Biology 17: e10396. 10.15252/msb.202110396 PMC8552907 34709727

[19] V'kovski, Philip , Markus Gerber , Jenna Kelly , Stephanie Pfaender , Nadine Ebert , Sophie Braga Lagache , Cedric Simillion , et al. 2019. “Determination of Host Proteins Composing the Microenvironment of Coronavirus Replicase Complexes by Proximity‐Labeling.” Elife 8: e42037. 10.7554/eLife.42037 PMC6372286 30632963

[20] Zhang, Yuehui , Limin Shang , Jing Zhang , Yuchen Liu , Chaozhi Jin , Yanan Zhao , Xiaobo Lei , et al. 2022. “An Antibody‐Based Proximity Labeling Map Reveals Mechanisms of SARS‐CoV‐2 Inhibition of Antiviral Immunity.” Cell Chemical Biology 29: 5–18.e6. 10.1016/j.chembiol.2021.10.008 PMC8527578 34672954

[21] Fang, Jingru , Colette Pietzsch , George Tsaprailis , Gogce Crynen , Kelvin Frank Cho , Alice Y. Ting , Alexander Bukreyev , Juan Carlos de la Torre , and Erica Ollmann Saphire . 2022. “Functional Interactomes of the Ebola Virus Polymerase Identified By Proximity Proteomics in the Context of Viral Replication.” Cell Reports 38: 110544. 10.1016/j.celrep.2022.110544 PMC10496643 35320713

[22] Muthaiyan, Mathavan , Leimarembi Devi Naorem , Vassavi Seenappa , Shilpa Sri Pushan , and Amouda Venkatesan . 2021. “Ebolabase: Zaire Ebolavirus‐Human Protein Interaction Database for Drug‐Repurposing.” International Journal of Biological Macromolecules 182: 1384–1391. 10.1016/j.ijbiomac.2021.04.184 34015403

[23] Viodé, Arthur , Kinga K. Smolen , Benoit Fatou , Zainab Wurie , Patrick Van Zalm , Mandy Kader Konde , Balla Moussa Keita , et al. 2022. “Plasma Proteomic Analysis Distinguishes Severity Outcomes of Human Ebola Virus Disease.” mBio 13: e00567–00522. 10.1128/mbio.00567-22 PMC9239184 35446128

[24] Shabman, Reed S. , Daisy W. Leung , Joshua Johnson , Nicole Glennon , Erol E. Gulcicek , Kathryn L. Stone , Lawrence Leung , et al. 2011. “DRBP76 Associates With Ebola Virus VP35 and Suppresses Viral Polymerase Function.” The Journal of Infectious Disease 204(Suppl 3): S911–S918. 10.1093/infdis/jir343 PMC3218669 21987769

[25] Lu, Jianhong , Yonggang Qu , Yuliang Liu , Rakesh Jambusaria , Ziying Han , Gordon Ruthel , Bruce D. Freedman , and Ronald N. Harty . 2013. “Host IQGAP1 and Ebola Virus VP40 Interactions Facilitate Virus‐Like Particle Egress.” Journal of Virology 87: 7777–7780. 10.1128/JVI.00470-13 PMC3700276 23637409

[26] Takahashi, Kei , Peter Halfmann , Masaaki Oyama , Hiroko Kozuka‐Hata , Takeshi Noda , and Yoshihiro Kawaoka . 2013. “DNA Topoisomerase 1 Facilitates the Transcription and Replication of the Ebola Virus Genome.” Journal of Virology 87: 8862–8869. 10.1128/JVI.03544-12 PMC3754039 23658456

[27] Martin‐Serrano, Juan , Trinity Zang , and Paul D. Bieniasz . 2001. “HIV‐1 and Ebola Virus Encode Small Peptide Motifs That Recruit Tsg101 to Sites of Particle Assembly to Facilitate Egress.” Nature Medicine 7: 1313–1319. 10.1038/nm1201-1313 11726971

[28] Tabata, Keisuke , Masaru Arimoto , Masashi Arakawa , Atsuki Nara , Kazunobu Saito , Hiroko Omori , Arisa Arai , et al. 2016. “Unique Requirement for ESCRT Factors in Flavivirus Particle Formation on the Endoplasmic Reticulum.” Cell Reports 16: 2339–2347. 10.1016/j.celrep.2016.07.068 27545892

[29] Quinn, Kathrina , Melinda A. Brindley , Melodie L. Weller , Nikola Kaludov , Andrew Kondratowicz , Catherine L. Hunt , Patrick L. Sinn , et al. 2009. “Rho GTPases Modulate Entry of Ebola Virus and Vesicular Stomatitis Virus Pseudotyped Vectors.” Journal of Virology 83: 10176–10186. 10.1128/JVI.00422-09 PMC2747995 19625394

[30] Hoenen, Thomas , Allison Groseth , and Heinz Feldmann . 2019. “Therapeutic Strategies to Target the Ebola Virus Life Cycle.” Nature Reviews Microbiology 17: 593–606. 10.1038/s41579-019-0233-2 31341272

[31] Rizk, Maryan G. , Christopher F. Basler , and John Guatelli . 2017. “Cooperation of the Ebola Virus Proteins VP40 and GP(1,2) with BST2 to Activate NF‐κB Independently of Virus‐Like Particle Trapping.” Journal of Virology 91: e01308‐17. 10.1128/JVI.01308-17 PMC5660480 28878074

[32] Okumura, Atsushi , Paula M. Pitha , and Ronald N. Harty . 2008. “ISG15 Inhibits Ebola VP40 VLP Budding in an L‐Domain‐Dependent Manner by Blocking Nedd4 Ligase Activity.” Proceedings of the National Academy of Sciences 105: 3974–3979. 10.1073/pnas.0710629105 PMC2268823 18305167

[33] Zhou, Yadi , Yuan Hou , Jiayu Shen , Yin Huang , William Martin , and Feixiong Cheng . 2020. “Network‐Based Drug Repurposing for Novel Coronavirus 2019‐nCoV/SARS‐CoV‐2.” Cell Discovery 6: 14. 10.1038/s41421-020-0153-3 PMC7073332 32194980

[34] Zhou, Yadi , Yuan Liu , Shagun Gupta , Mauricio I. Paramo , Yuan Hou , Chengsheng Mao , Yuan Luo , et al. 2023. “A Comprehensive SARS‐CoV‐2‐human Protein‐Protein Interactome Reveals COVID‐19 Pathobiology and Potential Host Therapeutic Targets.” Nature Biotechnology 41: 128–139. 10.1038/s41587-022-01474-0 PMC9851973 36217030

[35] Brandt, Janine , Lisa Wendt , Bianca S. Bodmer , Thomas C. Mettenleiter , and Thomas Hoenen . 2020. “The Cellular Protein CAD Is Recruited into Ebola Virus Inclusion Bodies by the Nucleoprotein NP to Facilitate Genome Replication and Transcription.” Cells 9: 1126. 10.3390/cells9051126 PMC7290923 32370067

[36] Younan, Patrick , Mathieu Iampietro , Rodrigo I. Santos , Palaniappan Ramanathan , Vsevolod L. Popov , and Alexander Bukreyev . 2018. “Disruption of Phosphatidylserine Synthesis or Trafficking Reduces Infectivity of Ebola Virus.” The Journal of Infectious Disease 218: S475–S485. 10.1093/infdis/jiy489 PMC6249599 30289506

[37] Hill‐Batorski, Lindsay , Peter Halfmann , Gabriele Neumann , and Yoshihiro Kawaoka . 2013. “The Cytoprotective Enzyme Heme Oxygenase‐1 Suppresses Ebola Virus Replication.” Journal of Virology 87: 13795–13802. 10.1128/JVI.02422-13 PMC3838215 24109237

[38] Patrick Reid, St. , Amy C. Shurtleff , Julie A. Costantino , Sarah R. Tritsch , Cary Retterer , Kevin B. Spurgers , and Sina Bavari . 2014. “HSPA5 Is an Essential Host Factor for Ebola Virus Infection.” Antiviral Research 109: 171–174. 10.1016/j.antiviral.2014.07.004 25017472

[39] Meisen, W. Hans , Zahra Bahrami Nejad , Miki Hardy , Huiren Zhao , Oliver Oliverio , Songli Wang , Christopher Hale , et al. 2020. “Pooled Screens Identify GPR108 and TM9SF2 as Host Cell Factors Critical for AAV Transduction.” Molecular Therapy‐Methods & Clinical Development 17: 601–611. 10.1016/j.omtm.2020.03.012 PMC7139131 32280726

[40] Luteijn, Rutger D. , Ferdy van Diemen , Vincent A. Blomen , Ingrid G. J. Boer , Saravanan Manikam Sadasivam , Toin H. van Kuppevelt , Ingo Drexler , et al. 2019. “A Genome‐Wide Haploid Genetic Screen Identifies Heparan Sulfate‐Associated Genes and the Macropinocytosis Modulator TMED10 as Factors Supporting Vaccinia Virus Infection.” Journal of Virology 93: e02160‐18. 10.1128/JVI.02160-18 PMC6580964 30996093

[41] Tanaka, Atsushi , Uranan Tumkosit , Shota Nakamura , Daisuke Motooka , Natsuko Kishishita , Thongkoon Priengprom , Areerat Sa‐ngasang , et al. 2017. “Genome‐Wide Screening Uncovers the Significance of N‐Sulfation of Heparan Sulfate as a Host Cell Factor for Chikungunya Virus Infection.” Journal of Virology 91: e00432‐17. 10.1128/JVI.00432-17 PMC5469253 28404855

[42] Heaton, Brook E. , Edward M. Kennedy , Rebekah E. Dumm , Alfred T. Harding , Matthew T. Sacco , David Sachs , and Nicholas S. Heaton . 2017. “A CRISPR Activation Screen Identifies a Pan‐Avian Influenza Virus Inhibitory Host Factor.” Cell Reports 20: 1503–1512. 10.1016/j.celrep.2017.07.060 PMC5568676 28813663

[43] Chinn, Leslie W. , Minzhong Tang , Bailey D. Kessing , James A. Lautenberger , Jennifer L. Troyer , Michael J. Malasky , Carl McIntosh , et al. 2010. “Genetic Associations of Variants in Genes Encoding HIV‐dependency Factors Required for HIV‐1 Infection.” The Journal of Infectious Diseases 202: 1836–1845. 10.1086/657322 PMC3107555 21083371

[44] Carette, Jan E. , Matthijs Raaben , Anthony C. Wong , Andrew S. Herbert , Gregor Obernosterer , Nirupama Mulherkar , Ana I. Kuehne , et al. 2011. “Ebola Virus Entry Requires the Cholesterol Transporter Niemann‐Pick C1.” Nature 477: 340–343. 10.1038/nature10348 PMC3175325 21866103

[45] Zhao, Yuguang , Jingshan Ren , Karl Harlos , Daniel M. Jones , Antra Zeltina , Thomas A. Bowden , Sergi Padilla‐Parra , Elizabeth E. Fry , and David I. Stuart . 2016. “Toremifene Interacts with and Destabilizes the Ebola Virus Glycoprotein.” Nature 535: 169–172. 10.1038/nature18615 PMC4947387 27362232

[46] Schimmöller, Frauke , Elva Díaz , Bettina Mühlbauer , and Suzanne R. Pfeffer . 1998. “Characterization of a 76 Kda Endosomal, Multispanning Membrane Protein That Is Highly Conserved Throughout Evolution.” Gene 216: 311–318. 10.1016/s0378-1119(98)00349-7 9729438

[47] Perrin, Jackie , Magda Mortier , Anne‐Claire Jacomin , Perrine Viargues , Dominique Thevenon , and Marie‐Odile Fauvarque . 2015. “The Nonaspanins TM9SF2 and TM9SF4 Regulate the Plasma Membrane Localization and Signalling Activity of the Peptidoglycan Recognition Protein PGRP‐LC in *Drosophila* .” Journal of Innate Immunity 7: 37–46. 10.1159/000365112 PMC6950948 25139117

[48] Kuhn, Jens H. , Sheli R. Radoshitzky , Alexander C. Guth , Kelly L. Warfield , Wenhui Li , Martin J. Vincent , Jonathan S. Towner , et al. 2006. “Conserved Receptor‐Binding Domains of Lake Victoria Marburgvirus and Zaire Ebolavirus Bind a Common Receptor.” Journal of Biological Chemistry 281: 15951–15958. 10.1074/jbc.M601796200 16595665

[49] Davey, Robert , Callie Donahue , Aditi Kesari , Naveen Thakur , Ling Wang , Sarah Hulsey‐Stubbs , Caroline Williams , et al. 2025. “A Protein‐Proximity Screen Reveals Ebola Virus Co‐Opts the mRNA Decapping Complex Through the Scaffold Protein EDC4.” Nature Communications 16: 8485. 10.1038/s41467-025-63392-4 PMC12475165 41006235

[50] Tian, Songhai , Khaja Muneeruddin , Mei Yuk Choi , Liang Tao , Robiul H. Bhuiyan , Yuhsuke Ohmi , Keiko Furukawa , et al. 2018. “Genome‐Wide CRISPR Screens for Shiga Toxins and Ricin Reveal Golgi Proteins Critical for Glycosylation.” PLoS Biology 16: e2006951. 10.1371/journal.pbio.2006951 PMC6258472 30481169

[51] Bergeret, Evelyne , Jackie Perrin , Michael Williams , Didier Grunwald , Elodie Engel , Dominique Thevenon , Emmanuel Taillebourg , et al. 2008. “TM9SF4 Is Required for Drosophila Cellular Immunity Via Cell Adhesion and Phagocytosis.” Journal Of Cell Science 121: 3325–3334. 10.1242/jcs.030163 18796536

[52] Jangra, Rohit K. , Andrew S. Herbert , Rong Li , Lucas T. Jae , Lara M. Kleinfelter , Megan M. Slough , Sarah L. Barker , et al. 2018. “Protocadherin‐1 Is Essential for Cell Entry by New World Hantaviruses.” Nature 563: 559–563. 10.1038/s41586-018-0702-1 PMC6556216 30464266

[53] Pillay, S. , N. L. Meyer , A. S. Puschnik , O. Davulcu , J. Diep , Y. Ishikawa , L. T. Jae , et al. 2016. “An Essential Receptor for Adeno‐Associated Virus Infection.” Nature 530: 108–112. 10.1038/nature16465 PMC4962915 26814968

[54] Shang, Limin , Yuehui Zhang , Yuchen Liu , Chaozhi Jin , Yanan Zhao , Jing Zhang , Pei‐Hui Wang , and Jian Wang . 2022. “An Antibody‐Based Proximity Labeling Protocol to Identify Biotinylated Interactors of SARS‐CoV‐2.” STAR Protocols 3: 101406. 10.1016/j.xpro.2022.101406 PMC9057979 35611119

[55] Li, Ning , Songfeng Wu , Chengpu Zhang , Cheng Chang , Jiyang Zhang , Jie Ma , Liwei Li , et al. 2012. “PepDistiller: A Quality Control Tool to Improve the Sensitivity and Accuracy of Peptide Identifications in Shotgun Proteomics.” Proteomics 12: 1720–1725. 10.1002/pmic.201100167 22623377

[56] Chang, Cheng , Mansheng Li , Chaoping Guo , Yuqing Ding , Kaikun Xu , Mingfei Han , Fuchu He , and Yunping Zhu . 2019. “PANDA: A Comprehensive and Flexible Tool for Quantitative Proteomics Data Analysis.” Bioinformatics 35: 898–900. 10.1093/bioinformatics/bty727 PMC6394390 30816924

[57] Teo, Guoci , Guomin Liu , Jianping Zhang , Alexey I. Nesvizhskii , Anne‐Claude Gingras , and Hyungwon Choi . 2014. “SAINTexpress: Improvements and Additional Features in Significance Analysis of INTeractome Software.” Journal of Proteomics 100: 37–43. 10.1016/j.jprot.2013.10.023 PMC4102138 24513533

[58] Mellacheruvu, Dattatreya , Zachary Wright , Amber L. Couzens , Jean‐Philippe Lambert , Nicole A. St‐Denis , Tuo Li , Yana V. Miteva , et al. 2013. “The CRAPome: A Contaminant Repository for Affinity Purification‐Mass Spectrometry Data.” Nature Methods 10: 730–736. 10.1038/nmeth.2557 PMC3773500 23921808

[59] Yu, Guangchuang , Li‐Gen Wang , Yanyan Han , and Qing‐Yu He . 2012. “Clusterprofiler: An R Package for Comparing Biological Themes Among Gene Clusters.” Omics: A Journal of Integrative Biology 16: 284–287. 10.1089/omi.2011.0118 PMC3339379 22455463

[60] Yu, Guangchuang , and Qing‐Yu He . 2016. “ReactomePA: An R/Bioconductor Package for Reactome Pathway Analysis and Visualization.” Molecular BioSystems 12: 477–479. 10.1039/c5mb00663e 26661513

[61] Yu, Guangchuang , Fei Li , Yide Qin , Xiaochen Bo , Yibo Wu , and Shengqi Wang . 2010. “GOSemSim: An R Package for Measuring Semantic Similarity Among GO Terms and Gene Products.” Bioinformatics 26: 976–978. 10.1093/bioinformatics/btq064 20179076

[62] Wang, Mingcong , Christina J. Herrmann , Milan Simonovic , Damian Szklarczyk , and Christian von Mering . 2015. “Version 4.0 of PaxDb: Protein Abundance Data, Integrated Across Model Organisms, Tissues, and Cell‐Lines.” Proteomics 15: 3163–3168. 10.1002/pmic.201400441 PMC6680238 25656970

[63] Sanjana, Neville E. , Ophir Shalem , and Feng Zhang . 2014. “Improved Vectors and Genome‐Wide Libraries for CRISPR Screening.” Nature Methods 11: 783–784. 10.1038/nmeth.3047 PMC4486245 25075903

[64] Xu, Wang , Yuhang Wang , Letian Li , Xiaoyun Qu , Quan Liu , Tiyuan Li , Shipin Wu , et al. 2022. “Transmembrane Domain of IFITM3 Is Responsible for Its Interaction With Influenza Virus HA(2) Subunit.” Virologica Sinica 37: 664–675. 10.1016/j.virs.2022.07.002 PMC9583175 35809785

[65] Wu, Xi , Nana Fang , Ziteng Liang , Jianhui Nie , Sen Lang , Changfa Fan , and Chunnan Liang , et al. 2023. “Development of a Bioluminescent Imaging Mouse Model for SARS‐CoV‐2 Infection Based on a Pseudovirus System.” Vaccines 11: 1133. 10.3390/vaccines11071133 PMC10385336 37514949

