# Supplementary material for: A multiplex interactome of Ebola virus proteins reveals TM9SF2 as a cell‐surface attachment factor that promotes viral entry
Source: IMetaOmics. 2026 Jul 2;3(3):e70116. doi: 10.1002/imo2.70116 (PMC13674394; doi:10.1002/imo2.70116)
Supplement: Supplementary file 1 [file IMO2-3-e70116-s002.docx]

**Supporting information to**

**A multiplex interactome of Ebola virus proteins reveals TM9SF2 as a cell-surface attachment factor that promotes viral entry**

Running title: TM9SF2 Facilitates EBOV Entry

Limin Shang^1^ | Shouwen Du^2,3^ | Yuchen Liu^1^ | Letian Li^4^ | Xi Wu^5^ | Yanzhi Yuan^1^ | Chaozhi Jin^1^ | Qingkun Mi^1^ | Yuhang Wang^3^ | Xiaoshuang Shi^4^ | Jiamin Wang^2,4^ | Guoqing Zhang^4^ | Yidan Ma^4^ | Weijin Huang^5^ | Jigang Wang^3^ | Jian Wang^1^ | Chang Li^4^

^1^State Key Laboratory of Medical Proteomics, Beijing Proteome Research Center, National Center for Protein Sciences (Beijing), Beijing Institute of Lifeomics, Beijing, China

^2^Key Laboratory of Livestock Disease Prevention of Guangdong Province, Key Laboratory for prevention and control of Avian Influenza and Other Major Poultry Diseases, Ministry of Agriculture and Rural Affairs, Institute of Animal Health, Guangdong Academy of Agricultural Sciences, Guangzhou, China

^3^Department of Infectious Diseases, Shenzhen People's Hospital, The First Affiliated Hospital, Southern University of Science and Technology, Shenzhen, China

^4^Changchun Veterinary Research Institute, Chinese Academy of Agricultural Sciences, State Key Laboratory of Pathogen and Biosecurity, Research Unit of Key Technologies for Prevention and Control of Virus Zoonoses, Chinese Academy of Medical Sciences, Changchun, China

^5^Division of HIV/AIDS and Sex-transmitted Virus Vaccines, National Institutes for Food and Drug Control, Beijing, China

Limin Shang, Shouwen Du, Yuchen Liu, Letian Li and Xi Wu contributed equally to this study.

*Correspondence

Chang Li, Changchun Veterinary Research Institute, Chinese Academy of Agricultural Sciences, State Key Laboratory of Pathogen and Biosecurity, Research Unit of Key Technologies for Prevention and Control of Virus Zoonoses, Chinese Academy of Medical Sciences, Changchun 130122, China. Email: [lichang78@163.com](mailto:lichang78@163.com)

Jian Wang, State Key Laboratory of Medical Proteomics, Beijing Proteome Research Center, National Center for Protein Sciences (Beijing), Beijing Institute of Lifeomics, Beijing 102206, China. Email: [wangjian@bmi.ac.cn](mailto:wangjian@bmi.ac.cn)

Jigang Wang, Department of Infectious Diseases, Shenzhen People's Hospital, The First Affiliated Hospital, Southern University of Science and Technology, Shenzhen 518020, China. Email: [jgwang@icmm.ac.cn](mailto:jgwang@icmm.ac.cn)

Weijin Huang, Division of HIV/AIDS and Sex-transmitted Virus Vaccines, National Institutes for Food and Drug Control, Beijing 102629, China. Email: [huangweijin@nifdc.org.cn](mailto:huangweijin@nifdc.org.cn)

**Figure S1.** Confocal imaging of EBOV proteins with or without TurboID tags. HEK293T cells co-expressing EBOV proteins with or without TurboID tags were fixed and stained using anti-Myc or anti-Flag antibodies to visualize the subcellular localization of TurboID-tagged and untagged EBOV proteins.

**Figure S2.** Proximity labeling of EBOV proteins in HEK293T cells. HEK293T cells expressing TurboID-tagged EBOV proteins were incubated with biotin, fixed, and stained with streptavidin-Alexa Fluor 568 (streptavidin-568) to detect biotinylated proteins, and anti-Myc antibody to detect EBOV-TurboID fusion proteins. Cells transfected with empty TurboID vector were used as a negative control.

**Figure S3.** Correlation, reproducibility, and sensitivity analyses of interactome datasets generated by Flag AP-MS, Strep AP-MS, and TurboID proximity labeling. Pearson correlation coefficients and heatmaps were generated for protein spectral counts from AP-MS or proximity labeling experiments using Flag-tagged (A, B), Strep-tagged (C, D), and TurboID-tagged (E, F) EBOV proteins. (G) Quantitative reproducibility between Flag and Strep AP-MS. Scatter plot showing Spearman correlation of spectral counts for interactions detected by both tags (r = 0.824, n = 1,728 PPIs). (H) Method combination sensitivity analysis. Bar plot showing the number of interactions retained under different method support definitions (≥1, ≥2, Flag∩Strep, includes TurboID, all three). (I) BFDR threshold sensitivity analysis. Bar plot showing the number of interactions retained for each method under different statistical thresholds (BFDR ≤0.05, BFDR ≤0.01, and SAINT ≥0.9 & BFDR ≤0.01).

**Figure S4.** Core EBOV-host PPI network. Network consisting of 1728 interactions between nine EBOV proteins and 914 host proteins. EBOV proteins are shown as diamonds; host proteins as circles.

**Figure S5.** Comparison of EBOV-host interactome with published dataset. (A) Venn diagram showing overlap between this study and two published EBOV PPI datasets [1, 2]. (B) Overlap of host factors with differentially expressed plasma proteomic dataset [3]. (C) Subnetwork of EBOV proteins and 17 overlapping host proteins in (B). Node color represents the fold change in protein abundance as reported in the proteomic dataset [3]. (D) Subnetwork between EBOV proteins and 10 known infection-associated host factors.

**Figure S6.** Subnetwork of Reactome pathway “RHO GTPase Effectors”. Network of EBOV proteins and host proteins involved in RHO GTPase signaling.

**Figure S7.** Functional enrichment analysis of host interactors for each EBOV protein. Gene Ontology (GO) and Reactome pathway enrichment analyses were performed on the host proteins of each EBOV protein.

**Figure S8.** Generation of *TM9SF2* and *NPC1* knockout cells for EBOV and MARV pseudovirus infection assays. (A) Four cell lines were infected with EBOVpv, and viral susceptibility was assessed. (B, C) Western blot analysis confirming the knockout of endogenous *TM9SF2* (B) and *NPC1* (C) in HEK293 cells. GAPDH was used as a loading control. (D) *NPC1* KO or wild-type HEK293 cells were infected with EBOV-VSVpv or MARV-VSVpv, and viral infectivity was quantified by luminescence intensity. (E) *TM9SF2* KO HEK293 cells and *TM9SF2* KD A549 cells, along with wild-type controls, were infected with MARV-VSVpv. Viral infectivity was determined by measuring luminescence intensity. Data are presented as mean ± SD. Statistical significance was assessed using a two-tailed Student’s *t*-test (*****p* < 0.0001).

**Figure S9.** TM9SF2 promotes EBOV infection via adhesion**.** (A) Schematic representation of the topological structures of EBOV GP and TM9SF2. SP, signal peptide; IFL, internal fusion loop; HR1/HR2, heptad repeat 1/2; TM, transmembrane domain; NTD, N-terminal domain. (B) Western blot of purified GP1, RBD and TM9SF2_ECD_ fragments tagged with Fc or His. SP, signal peptide. (C) Schematic of binding assay to detect interaction between of purified RBD-Fc and HEK293 cells. (D) ELISA-based inhibition assay using anti-TM9SF2 antibody to block GP1-TM9SF2_ECD_ interaction (n.s, not significant).

**Figure S10.** Flow cytometry analysis of RBD-Fc binding to wild-type and *TM9SF2* KO HEK293 cells. Purified RBD-Fc protein was incubated with wild-type HEK293 and *TM9SF2* KO cells, followed by staining with PE-conjugated goat anti-human IgG. Fluorescence signals were analyzed by flow cytometry. Fc protein alone was used as a negative control.

**Figure S11.** TM9SF2-dependent binding of RBD-Fc in WT, KO, and rescued HEK293 cells. Flow cytometry analysis of RBD-Fc binding in wild-type, *TM9SF2* KO, and TM9SF2-overexpressing HEK293 cells. Empty vector was used as control.

**Figure S12.** Construction and genotyping of *Tm9sf2*^+/-^ mice. (A) Schematic illustration of *Tm9sf2*^+/-^ mouse generation using CRISPR-Cas9 technology. Two sgRNAs targeting sites between exon 6 and 9 (spanning ~3.5 kb) are indicated by red triangles. Protospacer adjacent motif (PAM) sequences are highlighted in red. (B) Diagram showing positions of PCR primers (black arrows; F, forward; R, reverse) used for genotyping. Expected PCR product sizes for wild-type and KO alleles are listed. (C) PCR genotyping confirmed the 619 bp knockout allele in *Tm9sf2*^+/-^ mice, while the 4153 bp wild-type fragment couldn’t be amplified under PCR conditions for the knockout allele. NC, negative control. (D) Sanger sequencing results of PCR products from the *Tm9sf2*^+/-^ mice confirming the junction between exon 5 and exon 9, indicative of successful deletion.

**REFERENCES**

1. Batra, Jyoti, Judd F. Hultquist, Dandan Liu, Olena Shtanko, John Von Dollen, Laura Satkamp, Gwendolyn M. Jang, et al. 2018. “Protein Interaction Mapping Identifies RBBP6 as a Negative Regulator of Ebola Virus Replication.” *Cell* 175: 1917–1930 e1913. <https://doi.org/10.1016/j.cell.2018.08.044>

2. Muthaiyan, Mathavan, Leimarembi Devi Naorem, Vassavi Seenappa, Shilpa Sri Pushan, and Amouda Venkatesan. 2021. “Ebolabase: Zaire ebolavirus-human protein interaction database for drug-repurposing.” *International Journal of Biological Macromolecules* 182: 1384–1391. <https://doi.org/10.1016/j.ijbiomac.2021.04.184>

3. Viodé, Arthur, Kinga K. Smolen, Benoit Fatou, Zainab Wurie, Patrick Van Zalm, Mandy Kader Konde, Balla Moussa Keita, et al. 2022. “Plasma Proteomic Analysis Distinguishes Severity Outcomes of Human Ebola Virus Disease.” *mBio* 13: e00567–00522. <https://doi.org/doi:10.1128/mbio.00567-22>
